# Types of tobacco consumption and the oral microbiome in the United Arab Emirates Healthy Future (UAEHFS) Pilot Study

**DOI:** 10.1038/s41598-018-29730-x

**Published:** 2018-07-27

**Authors:** Yvonne Vallès, Claire K. Inman, Brandilyn A. Peters, Raghib Ali, Laila Abdel Wareth, Abdishakur Abdulle, Habiba Alsafar, Fatme Al Anouti, Ayesha Al Dhaheri, Divya Galani, Muna Haji, Aisha Al Hamiz, Ayesha Al Hosani, Mohammed Al Houqani, Abdulla Al Junaibi, Marina Kazim, Tomas Kirchhoff, Wael Al Mahmeed, Fatma Al Maskari, Abdullah Alnaeemi, Naima Oumeziane, Ravichandran Ramasamy, Ann Marie Schmidt, Michael Weitzman, Eiman Al Zaabi, Scott Sherman, Richard B. Hayes, Jiyoung Ahn

**Affiliations:** 1 Public Health Research Center, New York University Abu Dhabi, Abu Dhabi, UAE; 2 Department of Population Health, New York University School of Medicine, New York, USA; 3 Pathology and Laboratory Medicine Institute, Cleveland Clinic, Abu Dhabi, UAE; 4 Center for Biotechnology, Khalifa University of Science and Technology, Abu Dhabi, UAE; 5 Biomedical Engineering Department, Khalifa University of Science and Technology, Abu Dhabi, UAE; 6 College of Natural and Health Sciences, Zayed University, Abu Dhabi, UAE; 7 Department of Nutrition, College of Food and Agriculture; UAE University, Al-Ain, UAE; 8 Department of Medicine, College of Medicine and Health Sciences, UAE University, Al-Ain, UAE; 9 Department of Pediatrics, Zayed Military Hospital, Abu Dhabi, UAE; 10 Department of Pathology, Sheikh Khalifa Medical City, Abu Dhabi, UAE; 11 Heart and Vascular Institute, Cleveland Clinic, Abu Dhabi, UAE; 12 Institute of Public Health, College of Medicine and Health Sciences, UAE University, Al-Ain, UAE; 13 Department of Cardiology, Zayed Military Hospital, Abu Dhabi, UAE; 14 Abu Dhabi Blood Bank, SEHA, Abu Dhabi, UAE; 15 Diabetes Research Program, Division of Endocrinology, Diabetes and Metabolism, Department of Medicine, New York University School of Medicine, New York, USA; 16 Department of Environmental Medicine, New York University School of Medicine, New York, USA; 17 Department of Pediatrics, New York University School of Medicine, New York, USA; 18 NYU Perlmutter Cancer Center, New York, USA

## Abstract

Cigarette smoking alters the oral microbiome; however, the effect of alternative tobacco products remains unclear. Middle Eastern tobacco products like dokha and shisha, are becoming globally widespread. We tested for the first time in a Middle Eastern population the hypothesis that different tobacco products impact the oral microbiome. The oral microbiome of 330 subjects from the United Arab Emirates Healthy Future Study was assessed by amplifying the bacterial *16S rRNA* gene from mouthwash samples. Tobacco consumption was assessed using a structured questionnaire and further validated by urine cotinine levels. Oral microbiome overall structure and specific taxon abundances were compared, using PERMANOVA and DESeq analyses respectively. Our results show that overall microbial composition differs between smokers and nonsmokers (p = 0.0001). Use of cigarettes (p = 0.001) and dokha (p = 0.042) were associated with overall microbiome structure, while shisha use was not (p = 0.62). The abundance of multiple genera were significantly altered (enriched/depleted) in cigarette smokers; however, only *Actinobacillus*, *Porphyromonas*, *Lautropia* and *Bifidobacterium* abundances were significantly changed in dokha users whereas no genera were significantly altered in shisha smokers. For the first time, we show that smoking dokha is associated to oral microbiome dysbiosis, suggesting that it could have similar effects as smoking cigarettes on oral health.

## Introduction

The human oral microbiome (OM) is the second most diverse and densely populated microbiome of the human body^1^. It plays key roles in human digestion, protection against pathogen colonization and nitrate reduction^2^ and may have a role in human health including cardiovascular disease and cancer^3,4^. We recently reported that cigarette smoking^5^ and alcohol^6^ are important determinants of the oral microbiome. In particular, cigarette smoking is a cause of oral dysbiosis, affecting microbial diversity and its functional potential^5,7^.

Despite a global decline in tobacco consumption, tobacco use is still rising in African and Eastern Mediterranean countries, which is a significant public health concern^8^. Although cigarettes account for much of this increase, part of the increase is related to the popularization of alternative tobacco products common in Middle Eastern countries, such as dokha^9^ and shisha^10^. Dokha is a blend of tobacco leaves, barks, herbs, dried fruits and/or flowers and spices, which is smoked using a specialized pipe (midwakh) and is known to contain a much higher nicotine content than cigarettes^11^. Shisha is a fruit flavored tobacco comprised of shredded tobacco leaves, glycerol and other additives, which is smoked using a waterpipe^12^. Alternative tobacco products such as dokha and shisha, are, like cigarettes, a source of nicotine and other toxic products; however, the effect of these different types of tobacco on the oral microbiome remains unclear.

We tested for the first time the hypothesis that the oral microbiome is differentially impacted by specific tobacco products commonly used in Middle Eastern countries. We compared the effects of cigarette, dokha and shisha use on community composition of the oral microbiome by high-throughput sequencing of the bacterial *16S* Ribosomal RNA (*16S rRNA*) gene in 330 participants from the “UAE Healthy Future” (UAEHF) pilot study^13^.

## Results

**Figure 1:**
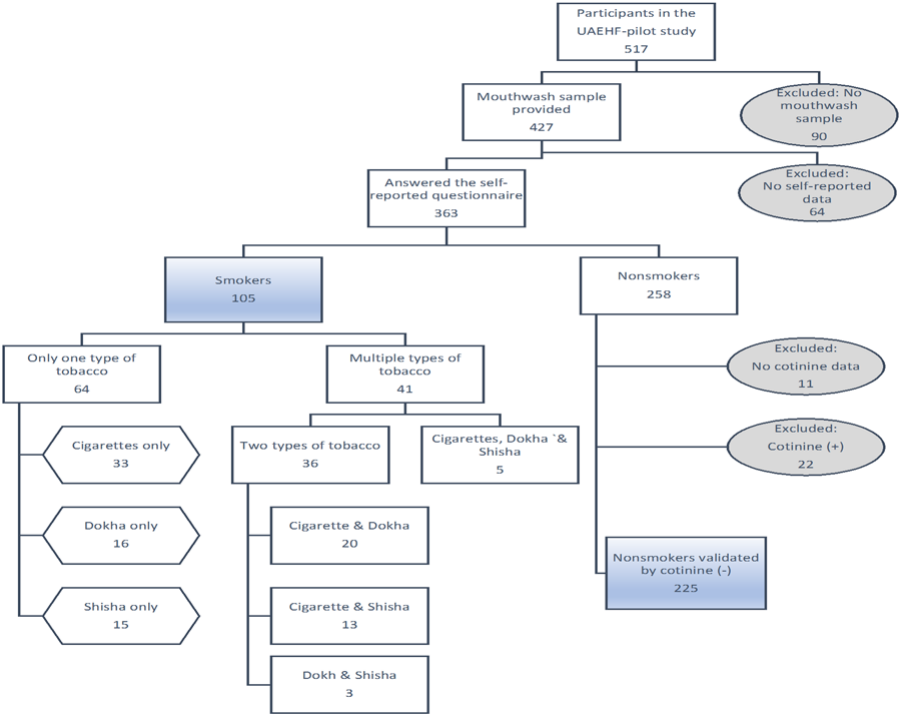
Flow chart depicting the classification of participants from the UAEHF pilot study. UAEHF pilot study participants were Emirati nationals aged 18 and above. Study participants completed a self-administered questionnaire including information on smoking habits. During the physical exam, participants provided blood, urine and mouthwash samples. From 517 consented study participants, 363 subjects completed the smoking section of the baseline questioners and provided mouthwash samples. A Cotinine test in urine was used to ascertain smoke exposure. These results were further used to validate the non-smoking self-reported data. We further excluded 33 subjects (11 had no cotinine data and 22 had self-reported as non-smokers but tested positive for cotinine). All individuals participating in the study read and signed an informed consent.

We studied 330 subjects, including 105 (31.8%) smokers and 225 nonsmokers (68.2%) (Fig 1, Table 1). Smokers were more likely to be men (96.2%), but other health related factors such as age, BMI and diabetic status (ascertained by HbA1c levels in blood), were not different between smokers and nonsmokers (p = 0.81, p = 0.11 and p = 0.13, respectively) with the exception of systolic blood pressure (p = 0.05). Among the 105 smokers, 39% smoked more than one tobacco product, with cigarettes most commonly used (67.6%), followed by dokha (42%) and shisha (34.3%). Participants that exclusively used cigarettes smoked an average of 9.2 cigarettes per day and exclusive dokha users smoked an average of 10.7 midwakh pipes per day. On the other hand, only 50% of the participants that used shisha exclusively smoked it on a daily basis, while 13.3% smoked it on a weekly (2–3 times per week) and 36.6% smoked it on a monthly basis.

**Table 1:** Characterization of smoking habits in the Emirati cohort.

	Total (n = 330)	Smokers (n = 105)	Nonsmokers (n = 225)	Cigarette (n = 33)	Dokha (n = 16)	Shisha (n = 15)	Cigarette & Dokha (n = 20)	Cigarette & Shisha (n = 13)	Dokha & Shisha (n = 3)	Cigarette, Dokha & Shisha (n = 5)
**Age**, mean (SD)	32.8 (10.3)	32.4 (9.6)	33.1 (10.8)	36.4 (11.4)	30.8 (7.5)	35.7 (10.6)	26.6 (5.3)	30.2 (7.1)	30 (7.8)	29.6 (8.2)
Sex, n (%)										
Female	104 (31.5)	4 (3.8)	100 (44.4)	2 (6.1)	0 (0)	1(6.7)	1 (5)	0 (0)	0 (0)	0 (0)
Male	226 (68.5)	101 (96.2)	125 (55.6)	31 (93.9)	16 (100)	14 (93.3)	19 (95)	13 (100)	3 (100)	5 (100)
BMI, mean (SD)	28.3 (6.5)	29.5 (6.9)	28.2 (6.2)	29.2 (6.8)	29.7 (6.6)	29.9 (5.5)	28 (6.8)	32 (9.1)	29.1 (12.4)	30.6 (3.7)
HbA1c, mean (SD)	5.6 (1.1)	5.7 (1.0)	5.6 (1.1)	5.9 (0.1)	5.9 (1.2)	5.7 (0.6)	5.7 (1.3)	5.4 (0.4)	5.4 (0.2)	5.5 (0.3)
Systolic BP mean (SD)	115.9 (16.4)	118.3 (15.9)	114.8 (16.1)	119.0 (15.5)	122.94 (15.6)	118.8 (12.4)	113.3 (19.9)	122.8 (16.0)	102.5 (9.2)	112.6 (3.4)

After data filtering, there were 16,132,922 high quality *16S rRNA* sequence reads ready for analysis for these study subjects (mean per subject: 48887.42; SD: 13,408.67). After low count filtering, the final data set was comprised of 13 phyla, 20 classes, 26 orders, 41 families, 57 genera, 26 species and 1,080 OTUs. We observed that amongst the 13 phyla, Firmicutes (50.0%), Bacteroidetes (21.7%), Proteobacteria (15.8%), Actinobacteria (6.7%) and Fusobacteria (4.7%) were the most abundant (Supplementary Table S1) and were present in all samples. Phyla such as Tenericutes, SR1 and Synergistetes, although in very low relative abundance, were present in more than 85% of the samples.

### Overall oral microbiome community

**Figure 2:**
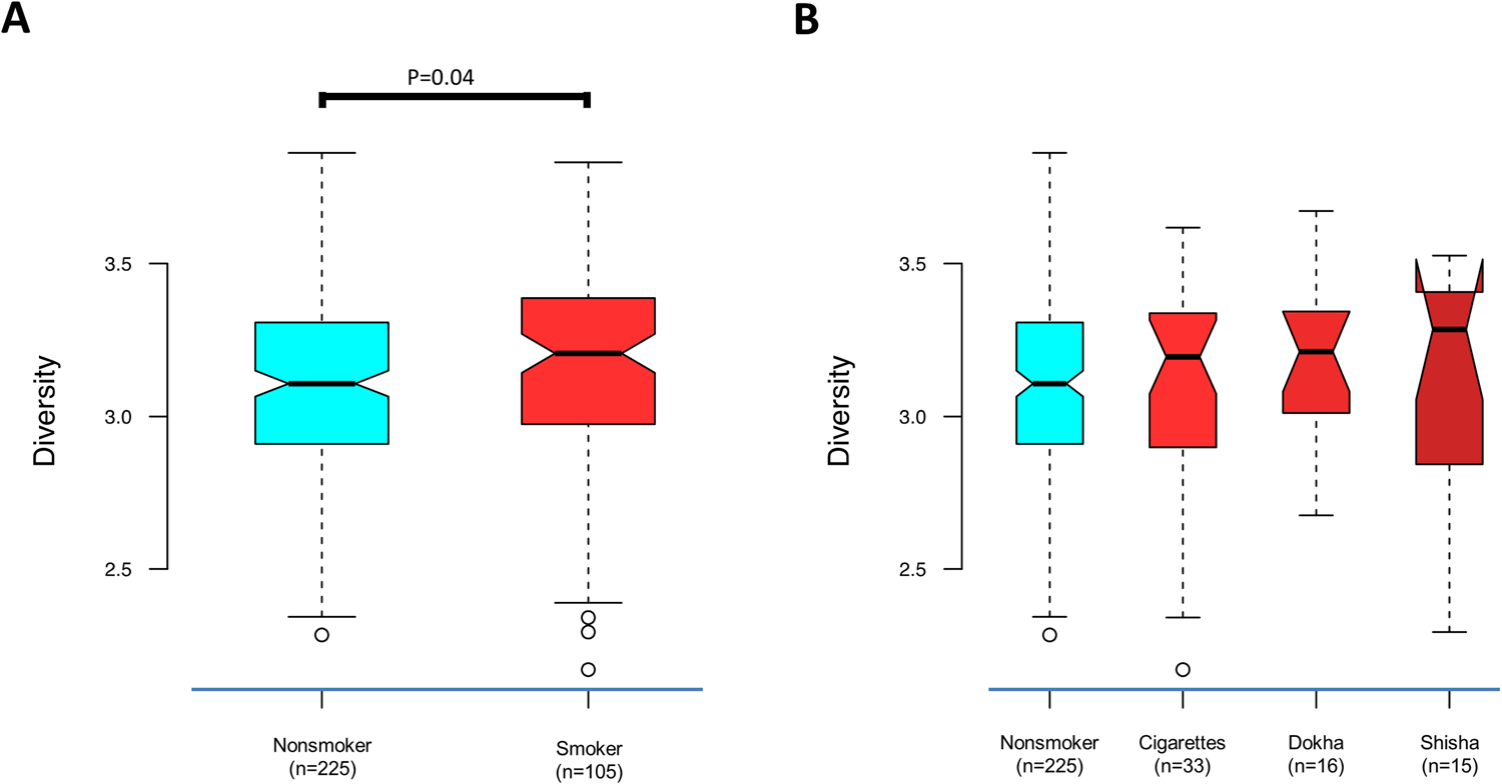
Characterization of the α-diversity of the Emirati oral microbiome. Diversity comparisons between (**A**) smokers (n = 105) and nonsmokers (n = 225) and (**B**) between tobacco types, cigarettes (n = 33), dokha (n = 16) and shisha (n = 15) versus nonsmokers (n = 225). Diversity was significantly greater in smokers than nonsmokers, but not when comparing single tobacco type use to nonsmokers. Only significant p values from linear regression are shown.

**Figure 3:**
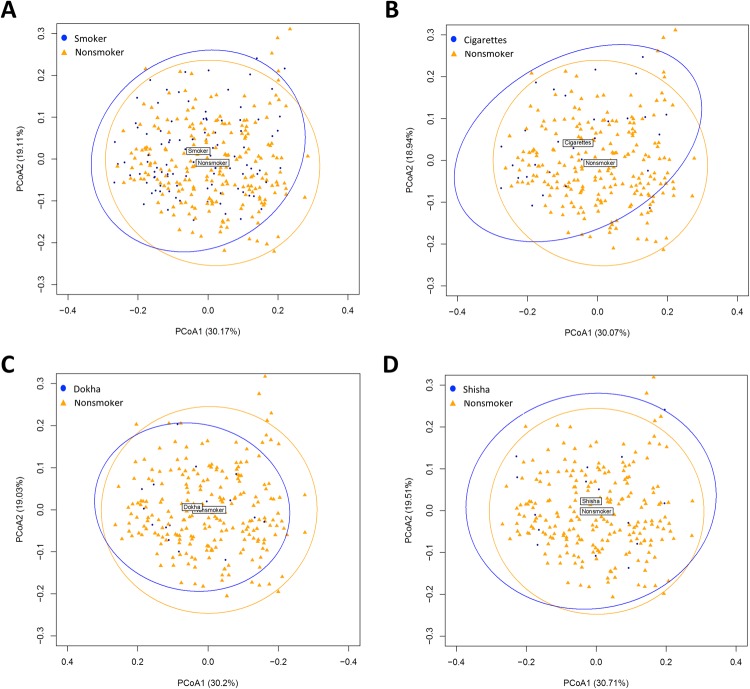
Principal Coordinate Analysis (PCoA) of the bacterial communities according to smoking use and tobacco types derived from Unifrac weighted distances. Significant differences between (**A**) smokers (n = 105) and nonsmokers (n = 225) were observed (p = 0.001), (**B**) cigarette (n = 33, p = 0.001) and (**C**) dokha smokers (n = 16, p = 0.042). However, no significant differences were identified between (**D**) shisha smokers (n = 15, p = 0.620) and nonsmokers. All nonsmoker participants were colored orange and all smokers independently of tobacco use or type in blue.

We found that microbial diversity (Shannon entropy) was marginally greater in all smokers compared with nonsmokers (p = 0.04, Fig. 2A); however, this was not observed when comparing single tobacco type users to nonsmokers (Fig. 2B). Based on Unifrac distance matrices, controlling for age, sex and batch effects, we found that the oral microbiome overall structure significantly differed between smokers and nonsmokers (p = 0.001, Fig. 3A). This finding was confirmed in the comparison of cotinine positive and cotinine negative participants (p = 0.001, Supplementary Figure S1), which was independent of their self-reported status. When considering single tobacco products independently, the oral microbiome structure of exclusively cigarette smokers (p = 0.001, Fig. 3B) and exclusively dokha users (p = 0.042, Fig. 3C) were significantly different from that of nonsmokers. However, exclusive shisha smokers’ oral microbiome was not significantly different from that of nonsmokers (p = 0.62, Fig. 3D). In addition, no significant differences were observed when comparing the overall oral microbiome structure amongst those who exclusively used one type of tobacco (cigarette, dokha and shisha smokers, p = 0.2; Supplementary Figure S2).

### Bacterial taxa abundance according to tobacco consumption and type

To determine the association of different tobacco products with oral bacterial taxa, we performed further detailed analyses.

#### Exclusively cigarette smokers (CS) vs. nonsmokers

**Figure 4:**
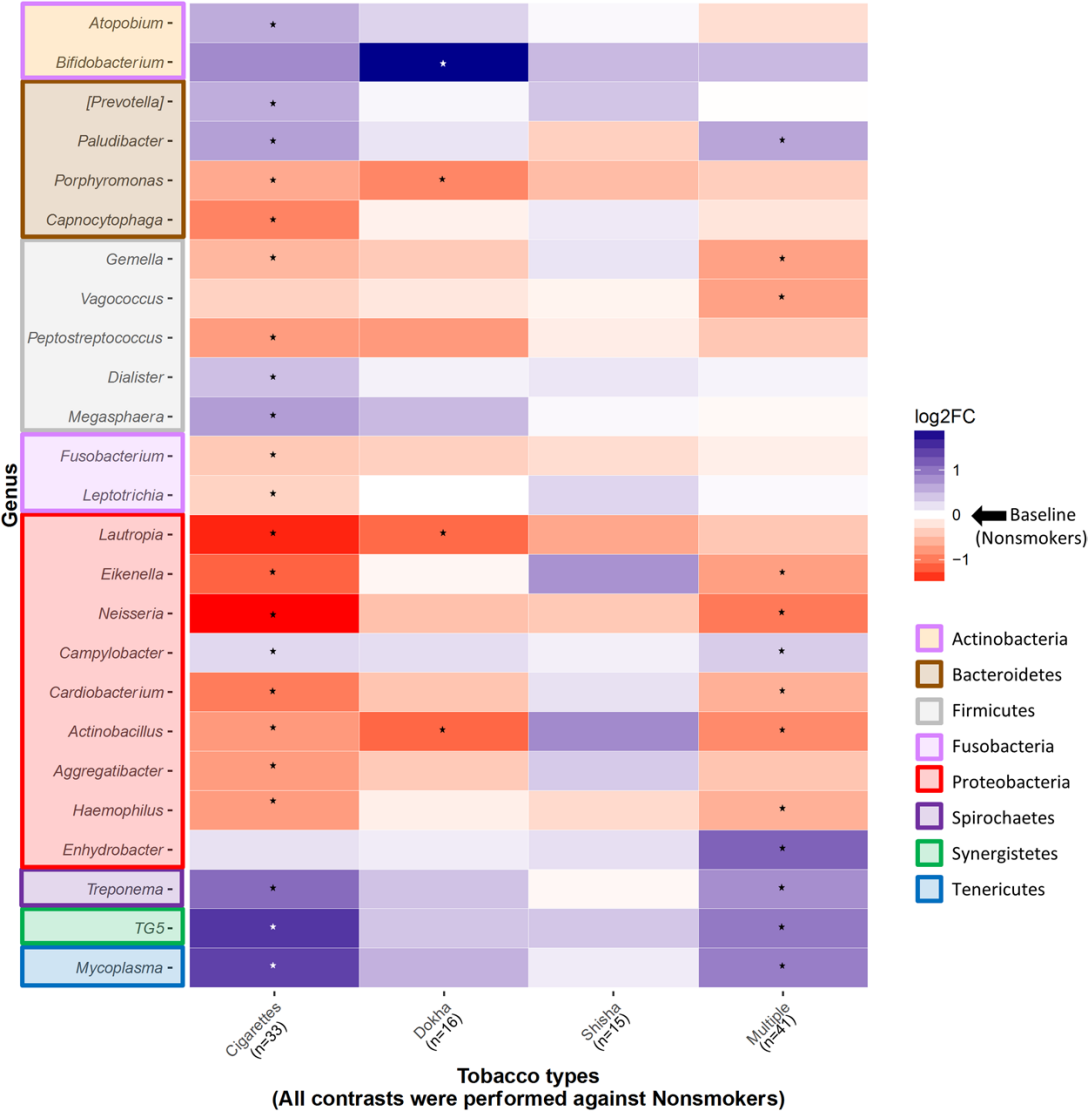
Log2 fold change of genera abundances in the oral microbiome relative to tobacco use. Heatmap of the genera that were in significantly different relative abundances when comparing nonsmokers (n = 225) to cigarette (n = 33), dokha (n = 16), shisha (n = 15) and multiple (n = 41) tobacco type smokers independently. All genera with q < 0.1 (indicated by stars) in at least one of the comparisons are shown. Heatmap displays log2 fold change when compared to nonsmokers.

Contrasts between CS (n = 33) and nonsmokers (n = 225) (Table 2, Supplementary Table S2, Fig. 4) showed that CS were depleted of the phylum Proteobacteria and in particular of its genera *Neisseria*, *Eikenella*, *Aggregatibacter*, *Actinobacillus*, *Haemophilus* and *Lautropia*, and the phylum Fusobacteria, represented at the genus level by *Fusobacterium* and *Leptotrichia*. Also, significantly depleted and not previously reported were the less abundant phyla SR1, GN02 and Cyanobacteria. In contrast, CS presented higher abundances at the phylum level of Spirochaetes, Synergistetes and Tenericutes being represented at the genus level by *Treponema*, TG5 and *Mycoplasma*, respectively. Furthermore, Firmicutes, Bacteroidetes and Actinobacteria were enriched at all lower taxonomical levels in CS, being characterized at the genus level among others by *Megasphaera* and *Dialister* (Firmicutes), *Paludibacter*, *Porphyromonas* and *Prevotella* (Bacteroidetes), and *Atopobium* (Actinobacteria).

**Table 2:** Differentially abundant taxa at selected taxonomical levels by type of tobacco use, compared to nonsmokers.

Taxa^a^	Non Smokers	Cigarettes			Dokha			Shisha			Multiple		
	Mean^b^	Mean^b^	Log2FC(95CI %)	q^c^	Mean^b^	Log2FC(95CI %)	q^c^	Mean^b^	Log2FC(95CI %)	q^c^	Mean^b^	Log2FC(95CI %)	q^c^
**Phylum**													
Cyanobacteria	5.76	0.83	−1.38 (−2.36, −0.39)	1.00E-02	0.00	−1.40 (−2.32, −0.48)	4.00E-02	1.31	−1.16 (−2.08, −0.25)	8.00E-02	0.90	−1.30 (−2.29, −0.31)	3.00E-02
Fusobacteria	1733.44	1254.80	−0.48 (−0.73, −0.23)	0.00E + 00	1788.84	0.08 (−0.26, 0.41)	8.50E-01	1770.45	0.02 (−0.32, 0.36)	1.00E + 00	1739.36	0.06 (−0.17, 0.30)	6.40E-01
GN02	3.27	0.52	−2.03 (−2.83, −1.22)	0.00E + 00	1.75	−0.86 (−1.74, 0.02)	2.50E-01	2.23	−0.83 (−1.72, 0.05)	2.80E-01	3.17	−0.35 (−1.10, 0.40)	5.90E-01
Proteobacteria	7106.72	3563.83	−1.08 (−1.44, −0.72)	0.00E + 00	6666.25	−0.20 (−0.67, 0.27)	7.60E-01	6525.77	−0.26 (−0.74, 0.22)	9.10E-01	4984.18	−0.56 (−0.90, −0.22)	1.00E-02
Spirochaetes	157.39	337.85	1.07 (0.52, 1.61)	0.00E + 00	236.53	0.68 (0.00, 1.36)	2.50E-01	122.97	−0.11 (−0.79, 0.58)	1.00E + 00	273.62	0.92 (0.40, 1.43)	1.00E-02
SR1	81.79	26.15	−1.31 (−2.01, −0.60)	0.00E + 00	84.05	−0.28 (−1.10, 0.54)	8.20E-01	28.36	−1.10 (−1.92, −0.27)	8.00E-02	73.22	−0.40 (−1.07, 0.27)	4.60E-01
Synergistetes	18.53	81.96	1.72 (1.09, 2.35)	0.00E + 00	23.42	0.66 (−0.11, 1.43)	3.00E-01	20.68	0.31 (−0.46, 1.09)	9.30E-01	36.90	1.03 (0.43, 1.64)	1.00E-02
Tenericutes	23.83	43.11	0.68 (0.06, 1.31)	5.00E-02	29.92	0.43 (−0.32, 1.19)	5.60E-01	16.83	−0.35 (−1.11, 0.42)	9.30E-01	30.30	0.47 (−0.12, 1.06)	3.10E-01
**Phylum; Class; Order; Family; Genus**													
Actinobacteria; Actinobacteria;Bifidobacteriales; Bifidobacteriaceae;Bifidobacterium	6.51	13.16	0.84 (0.03, 1.65)	1.00E-01	57.14	1.88 (1.01, 2.74)	1.17E-03	8.09	0.49 (−0.37, 1.36)	8.95E-01	8.81	0.49 (−0.29, 1.27)	4.45E-01
Actinobacteria; Coriobacteriia;Coriobacteriales; Coriobacteriaceae;Atopobium	142.80	221.05	0.59 (0.14, 1.05)	4.00E-02	181.90	0.31 (−0.27, 0.89)	6.74E-01	144.84	0.05 (−0.54, 0.64)	9.70E-01	117.81	−0.26 (−0.69, 0.17)	4.48E-01
Bacteroidetes; Bacteroidia;Bacteroidales; [Paraprevotellaceae];[Prevotella]	732.67	986.83	0.58 (0.18, 0.97)	2.00E-02	715.75	0.06 (−0.45, 0.58)	8.96E-01	856.09	0.41 (−0.11, 0.94)	8.95E-01	717.10	−0.01 (−0.38, 0.37)	9.64E-01
Bacteroidetes; Bacteroidia;Bacteroidales; Porphyromonadaceae;Paludibacter	23.68	38.59	0.68 (0.12, 1.24)	5.00E-02	22.67	0.19 (−0.50, 0.88)	7.83E-01	13.34	−0.37 (−1.07, 0.33)	8.95E-01	30.94	0.64 (0.11, 1.17)	8.04E-02
Bacteroidetes; Bacteroidia; Bacteroidales;Porphyromonadaceae; Porphyromonas	913.25	618.01	−0.67 (−1.07, −0.26)	1.00E-02	552.77	−0.93 (−1.45, −0.40)	1.44E-02	701.09	−0.53 (−1.06, 0.00)	7.36E-01	841.62	−0.39 (−0.77, −0.01)	1.58E-01
Bacteroidetes; Flavobacteriia; Flavobacteriales;Flavobacteriaceae; Capnocytophaga	157.25	75.70	−0.95 (−1.35, −0.54)	0.00E + 00	141.59	−0.11 (−0.63, 0.41)	8.29E-01	171.86	0.15 (−0.38, 0.68)	9.70E-01	130.23	−0.21 (−0.59, 0.17)	4.91E-01
Firmicutes; Bacilli; Gemellales;Gemellaceae;Gemella	23.11	16.75	−0.57 (−1.03, −0.12)	4.00E-02	18.68	−0.42 (−1.00, 0.15)	4.79E-01	29.34	0.19 (−0.39, 0.77)	9.70E-01	14.68	−0.73 (−1.16, −0.31)	1.68E-02
Firmicutes;Bacilli; Lactobacillales;Enterococcaceae; Vagococcus	1.25	0.91	−0.36 (−0.98, 0.25)	3.70E-01	1.27	−0.19 (−0.92, 0.54)	7.83E-01	1.25	−0.09 (−0.82, 0.65)	9.70E-01	0.71	−0.73 (−1.31, −0.15)	7.20E-02
Firmicutes; Clostridia; Clostridiales;Peptostreptococcaceae; Peptostreptococcus	95.48	54.49	−0.77 (−1.29, −0.24)	2.00E-02	53.88	−0.79 (−1.44, −0.14)	2.04E-01	95.87	−0.14 (−0.80, 0.52)	9.70E-01	74.96	−0.44 (−0.94, 0.05)	2.29E-01
Firmicutes; Clostridia; Clostridiales;Veillonellaceae; Dialister	54.07	63.92	0.45 (0.05, 0.84)	8.00E-02	47.16	0.10 (−0.42, 0.61)	8.29E-01	53.78	0.17 (−0.35, 0.69)	9.70E-01	46.95	0.08 (−0.30, 0.45)	8.29E-01
Firmicutes; Clostridia; Clostridiales;Veillonellaceae; Megasphaera	156.45	266.63	0.70 (0.19, 1.20)	3.00E-02	228.18	0.49 (−0.14, 1.12)	4.79E-01	160.45	0.05 (−0.59, 0.69)	9.70E-01	150.16	−0.05 (−0.53, 0.42)	8.60E-01
Fusobacteria; Fusobacteriia;Fusobacteriales; Fusobacteriaceae;Fusobacterium	989.40	743.50	−0.43 (−0.70, −0.16)	1.00E-02	747.78	−0.37 (−0.73, −0.01)	3.52E-01	822.11	−0.27 (−0.63, 0.10)	8.95E-01	890.29	−0.13 (−0.38, 0.12)	5.07E-01
Fusobacteria; Fusobacteriia;Fusobacteriales; Leptotrichiaceae; Leptotrichia	629.22	505.20	−0.35 (−0.66, −0.03)	9.00E-02	586.31	0.00 (−0.42, 0.42)	9.89E-01	777.87	0.29 (−0.14, 0.71)	8.95E-01	596.20	0.04 (−0.26, 0.33)	8.60E-01
Proteobacteria; Betaproteobacteria;Burkholderiales; Burkholderiaceae; Lautropia	232.27	68.79	−1.45 (−2.09, −0.81)	0.00E + 00	80.40	−1.12 (−1.88, −0.37)	5.08E-02	130.76	−0.69 (−1.45, 0.07)	7.36E-01	161.85	−0.44 (−1.05, 0.17)	3.58E-01
Proteobacteria; Betaproteobacteria;Neisseriales; Neisseriaceae; Eikenella	27.43	11.01	−1.16 (−1.67, −0.66)	0.00E + 00	26.74	−0.07 (−0.70, 0.55)	8.96E-01	54.70	0.78 (0.14, 1.41)	7.36E-01	15.48	−0.75 (−1.22, −0.27)	1.80E-02
Proteobacteria; Betaproteobacteria;Neisseriales; Neisseriaceae; Neisseria	2527.32	896.43	−1.51 (−2.03, −0.99)	0.00E + 00	2053.66	−0.49 (−1.14, 0.15)	4.79E-01	2184.49	−0.43 (−1.09, 0.22)	8.95E-01	1326.65	−1.02 (−1.51, −0.53)	2.59E-03
Proteobacteria; Epsilonproteobacteria;Campylobacterales; Campylobacteraceae; Campylobacter	142.45	158.50	0.27 (0.02, 0.52)	9.00E-02	143.80	0.24 (−0.09, 0.57)	4.79E-01	141.67	0.11 (−0.23, 0.45)	9.70E-01	156.84	0.36 (0.13, 0.59)	1.80E-02
Proteobacteria; Gammaproteobacteria;Cardiobacteriales; Cardiobacteriaceae; Cardiobacterium	23.01	9.58	−1.00 (−1.53, −0.46)	0.00E + 00	14.45	−0.46 (−1.12, 0.20)	4.79E-01	30.17	0.21 (−0.46, 0.87)	9.70E-01	11.18	−0.62 (−1.12, −0.12)	7.52E-02
Proteobacteria; Gammaproteobacteria;Pasteurellales; Pasteurellaceae; Actinobacillus	31.13	17.96	−0.79 (−1.41, −0.17)	4.00E-02	11.02	−1.14 (−1.88, −0.40)	4.83E-02	88.26	0.84 (0.09, 1.58)	7.36E-01	16.37	−0.90 (−1.49, −0.31)	1.98E-02
Proteobacteria; Gammaproteobacteria;Pasteurellales; Pasteurellaceae; Aggregatibacter	353.06	196.38	−0.77 (−1.25, −0.29)	1.00E-02	257.84	−0.42 (−1.03, 0.18)	4.79E-01	494.65	0.37 (−0.24, 0.98)	8.95E-01	255.76	−0.46 (−0.91, −0.01)	1.58E-01
Proteobacteria; Gammaproteobacteria;Pasteurellales; Pasteurellaceae; Haemophilus	3786.72	2365.12	−0.77 (−1.16, −0.38)	0.00E + 00	3404.50	−0.13 (−0.64, 0.37)	7.83E-01	3396.53	−0.29 (−0.81, 0.22)	8.95E-01	2333.62	−0.62 (−0.99, −0.26)	1.68E-02
Proteobacteria; Gammaproteobacteria;Pseudomonadales; Moraxellaceae; Enhydrobacter	2.18	2.25	0.21 (−0.71, 1.13)	8.10E-01	1.47	0.13 (−0.75, 1.01)	8.85E-01	1.91	0.22 (−0.65, 1.10)	9.70E-01	9.95	1.15 (0.23, 2.06)	7.20E-02
Spirochaetes; Spirochaetes; Spirochaetales;Spirochaetaceae; Treponema	161.93	346.73	1.08 (0.53, 1.63)	0.00E + 00	201.42	0.43 (−0.24, 1.11)	5.36E-01	131.28	−0.06 (−0.75, 0.62)	9.70E-01	264.57	0.81 (0.29, 1.33)	1.80E-02
Synergistetes; Synergistia; Synergistales;Dethiosulfovibrionaceae; TG5	18.16	64.52	1.50 (0.89, 2.12)	0.00E + 00	19.19	0.41 (−0.33, 1.15)	6.56E-01	23.06	0.40 (−0.35, 1.14)	8.95E-01	34.89	0.97 (0.38, 1.55)	1.68E-02
Tenericutes; Mollicutes; Mycoplasmatales;Mycoplasmataceae; Mycoplasma	14.78	40.78	1.41 (0.73, 2.09)	0.00E + 00	15.67	0.55 (−0.24, 1.34)	4.79E-01	11.52	0.14 (−0.66, 0.93)	9.70E-01	21.21	0.93 (0.28, 1.57)	3.31E-02

^a^Only those taxa that have a significantly differential abundance with q < 0.10 and a Cook’s distance < 10 in at least one contrast are shown.

^b^Mean values refer to mean normalized counts of taxa according to each group.

^c^FDR adjusted p value. FDR adjustment was implemented at each level independently (i.e. phylum, genus).

#### Exclusively dokha smokers (DS) vs. nonsmokers

Consistent with the patterns observed in CS, taxa dynamics in DS (n = 16) differed significantly from nonsmokers with the depletion of the phylum Cyanobacteria observed in CS, and in the genera *Actinobacillus*, *Lautropia* (Proteobacteria), and *Porphyromonas* (Bacteroidetes), which were also depleted in CS (Table 2, Supplementary Table S2, Fig. 4). In contrast, DS were exclusively enriched in the genus *Bifidobacterium* (Actinobacteria).

#### Exclusively shisha smokers (SS) vs. nonsmokers

Consistent with overall microbial composition comparisons, only four taxa were identified as having a significantly different relative abundance between SS (n = 15) and nonsmokers (Table 2, Supplementary Table S2, Fig. 4). The phyla Cyanobacteria and SR1 and the classes Chloroplast (Cyanobacteria) and BD1-5 (GN02) were all significantly depleted in SS when compared to nonsmokers.

#### Multiple tobacco type smokers (MS) vs. nonsmokers

We also performed the contrast between MS (n = 41) against nonsmokers (Table 2, Supplementary Table S2, Fig. 4). Depletion and enrichment patterns of taxa relative abundances were for the most part mirroring those observed in the contrast between CS and nonsmokers with some exceptions, in particular, the significant enrichment in MS of the genera *Campylobacter* and *Enhydrobacter* (Proteobacteria), and the depletion of the genus *Vagococcus* (Firmicutes); the two latter genera were not observed in other contrasts.

#### Contrasts between tobacco types

Comparisons between CS and DS revealed no significant differences at any taxonomical level in the taxa relative abundances of their oral microbiome (Supplementary Table S3). Comparisons between CS and SS however showed similar results to those observed in the contrast between CS and nonsmokers, with patterns for SS similar to that of nonsmokers. Only the depletion of the genus *Actinobacillus* was consistently observed in cigarette vs. shisha users and dokha vs. shisha smokers.

## Discussion

This study encompasses the first characterization of the oral microbiome of the Emirati population and describes, for the first time, the specific effects on oral bacterial community structure of two regional products, dokha and shisha, with the latter experiencing increased worldwide usage in recent years^14^. We found that the Emirati population exhibited a diverse oral microbiome and that overall microbial diversity and composition were associated with use of tobacco products (Fig. 4). In particular, smoking in general, exclusive use of cigarettes and exclusive use of dokha were associated with significant alterations of oral microbiome structure and relative taxa abundances. Exclusive use of shisha was not associated with alterations in overall microbiome structure; however, depletion was noted in phyla Cyanobacteria and SR1, and classes Chloroplast and BD1-5.

The oral microbiome of the Emirati population presented a composition similar to that of other populations in the United States^5,15^, Japan and Korea^16,17^, China^18^, and among Amazonian Amerindians^19,20^, characterized by community dominance of phyla Firmicutes, Bacteroidetes, Proteobacteria, Actinobacteria and Fusobacteria and genera *Streptococcus*, *Prevotella*, *Haemophilus*, *Veillonella* and *Neisseria*. It would appear that the oral microbiome tends to have a generally similar community structure globally, despite there being wide differences in lifestyle and oral hygiene practices between populations. Of course, at a further detailed level of analysis between populations, there may be traits that tend to be more population-specific. For example, SR1 was reported as part of the core microbiome only in a Saudi Arabian population^21^. In the Emirati population SR1 was observed in 87% of the samples. This high carriage could be a characteristic of Middle Eastern populations, requiring further exploration. Further detailed analyses involving for example transcriptomics, metabolomics or proteomics could potentially reveal further population-specific biomarkers.

We found that tobacco use in general, was marginally associated (p = 0.04), with greater diversity of the oral microbiome. Similar associations have been reported in other studies^16,22,23^. However, this is not consistently observed with some reporting no change in diversity^24–26^. Exposure to tobacco smoke results in functional and structural changes in saliva and the oral environment^27–29^, which may impact on reduced immune fitness and decreased ability of the autochthonous bacteria to compete with transient taxa for nutrients; this may result in the increased diversity of the OM^7,30^ observed in smokers. Perhaps of clinical importance, periodontal disease and gingivitis, which are known to be associated with tobacco use, are also characterized by a higher diversity of the OM^7,22,31–33^, potentially indicating an early pathway to oral disease in smokers.

Consistent with previous studies, we found that smokers were significantly depleted of Proteobacteria such as *Neisseria*, *Haemophilus* and *Lautropia*)^5,23,30^, and enriched of *Bifidobacterium*^5^ and TG5^34^. In addition, we reported for the first time the depletion in all smokers of the less abundant phylum SR1 (Supplementary Table S4). Members of the SR1 phylum are predominantly uncultivated, non-respiring oral bacteria that possess an altered genetic code, where the usual UGA stop codon is reassigned to a glycine)^35–37^. This change in the genetic code of SR1 may limit synergistic relationships within the oral bacterial community^36^, which is potentially related to the depleted relative abundances we observed.

Exclusive cigarette use was also associated with differentials in specific oral taxa, including a wider range of taxa than that found for all smokers combined. CS were depleted when compared to nonsmokers in the genera *Aggregatibacter* (Proteobacteria), *Capnocytophaga* and *Porphyromonas* (Bacteroidetes) and in particular of the phylum Fusobacteria represented by significantly depleted *Fusobacterium* and *Leptotrichia*. We previously reported^5^ similar differentials and related the bacterial genes involved to xenobiotic metabolism of toluene, in agreement with Peralbo-Molina, *et al*.^38^ who observed that exhaled breath condensate of cigarette smokers contained lower levels of p-cresol, a toluene metabolite. We also found that CS were significantly enriched in *Atopobium* and *Bifidobacterium* (Actinobacteria), TG5 (Synergistetes), *Treponema* (Synergistetes), *Campylobacter* and *Eikenella* (Proteobacteria) and *Megasphaera* (Firmicutes) consistent overall with previous results^5,30,34,39,40^.

We report for the first time on the impact of dokha and shisha on the oral microbiome. Dokha was associated with similar patterns of OM dysbiosis as found for cigarette use, although significant associations were found for fewer taxa, among which, the depletion of the phylum Cyanobacteria, the genera *Actinobacillus*, *Lautropia* (Proteobacteria) and *Porphyromonas* (Bacteroidetes) and the enrichment of the genus *Bifidobacterium* (Actinobacteria). The lower number of significant associations of taxa differentials with the use of dokha is probably due to the low number of participants (n = 16) that exclusively smoked dokha. Dokha is commonly consumed in the UAE using the traditional  midwakh pipe. Midwakh users in the UAE consume dokha on average 12 times per day, being equivalent to smoking 6 grams of dokha per day^9^, which is reflected on its effects on the oral microbiome. Dokha toxicants and health effects have received limited study, although notably Shaikh *et al*. reported increased systolic blood pressure, heart rate and respiratory rate in users^41^, indicating that these exposures constitute a potentially significant, yet understudied threat to health in the Middle Eastern region.

Although we found that shisha use was not related to overall oral microbiome structural changes, taxa relative abundance analysis identified the phyla Cyanobacteria and SR1, and the classes Chloroplast and BD1-5 as significantly depleted in shisha smokers when compared to nonsmokers, The lack of significance observed for the majority of the taxa is likely due to both the low n number available (n = 15) for exclusive shisha users as well as to the infrequency of shisha use, rather than the absence of toxicants in this product^12,42^. Shisha is usually associated with social gatherings, and consumption is often on a weekly to monthly basis^43^. As the OM is resilient^44^ and smoking related changes may not be permanent if cessation occurs^5^, the frequency with which participants smoke shisha could partially explain the patterns observed. Shisha smoking has been associated with esophageal squamous cell carcinoma^45^, low birth weight of infants from smoking mothers^46^ and cardiovascular effects^47^, and hence warrants for further study.

Users of multiple tobacco types in our study tended to show similar depletion/enrichment patterns of taxa relative abundances as cigarette smokers, largely because cigarette use was the most common tobacco use type in this group. Potentially of note, increased abundance of *Enhydrobacter* was related to joint use of cigarettes and dokha. This bacterium grows in the presence of ammonia^48^ which is believed to be common in both these products. In the case of cigarettes, ammonia is added to facilitate freeing of nicotine molecules by raising pH^49^.

This investigation is the largest study of the oral microbiome of an Arabic population. In contrast to most studies that rely exclusively on self-reported questionnaire, we validated nonsmoking status by urinary cotinine measurement. Although associations were identified for the tobacco types commonly used in this region, larger studies, which would provide stronger statistical power, with more detailed information on tobacco use patterns and frequency will be needed to further delineate differentials in tobacco products and the oral microbiome. We are currently recruiting participants to the UAEHFS to address this and other health-related issues for the UAE population. Although amplicon pyrosequencing has major advantages for human microbiome studies, it has also some limitations, such as the possible overestimation of OTU richness due to homopolymer errors (repeated nucleotides), inaccuracies of taxonomic identification due to the short length of the sequences and the introduction of primer and sequencing related biases^50–52^. While our research using the *16 S rRNA* sequencing approach was appropriate for identifying taxonomies, future studies should investigate functional capacity of the microbiome, using full shotgun metagenomics sequencing and other methodologies.

In summary, we characterized the oral microbiome in the Emirati population and found that tobacco use had an important impact on the oral microbiome, particularly with regard to cigarette and dokha use. The abundance of multiple taxa and in particular that of 15 genera was significantly altered (enriched or depleted) in cigarette smokers; however, at the genus level, only the abundance of *Actinobacillus*, *Lautropia*, *Porphyromonas* and *Bifidobacterium* were significantly altered in users of dokha, and none were observed in shisha smokers. Our results suggest that cigarettes and other local tobacco products alter the oral microbiome structure and specific taxa abundance in the Emirati population.

## Methods

### Study Population

UAEHF pilot study participants were recruited in a 5-month period between December 2014 and April 2015 at Zayed Military Primary Health Clinic (ZMH PHCC) and Abu Dhabi Blood Bank (ADBB), both of which are licensed for clinical research by the Health Authority of Abu Dhabi. Eligible Emirati nationals (aged 18 and above) completed a self-administered questionnaire including information on socio-demographic factors, lifestyle and medical history. Study participants completed physical and clinical exams, including measurements of anthropometry, body composition, and blood pressure^13^. During the physical exam, participants also provided blood, urine and mouthwash samples. From 517 consented study participants, 363 subjects completed baseline questionnaires and provided mouthwash samples. We further excluded 33 subjects who had inconsistent smoking data (see smoking definition below). Therefore, our analytic dataset was comprised of 330 subjects (Fig. 1). This study was approved by the Institutional Review Boards (IRB) of Sheikh Khalifa Medical City (SKMC), Zayed Military Hospital (ZMH), New York University Abu Dhabi (NYUAD) and NYU Langone Medical Center, New York. All individuals participating in the study read and signed an informed consent. All experiments were performed in accordance with relevant guidelines and regulations.

### Measurements

#### Definition of smoking

Detailed information on cigarette smoking, including smoking status, tobacco type used and smoking history, was ascertained by questionnaire. We also measured cotinine in urine by COT rapid test cassette (International Biomedical Supplies), with a cut off concentration of 200 ng/ml for tobacco smoke exposure. We further excluded from analysis 22 subjects with positive cotinine test who had self-reported as nonsmokers and 11 subjects with missing cotinine data. Tobacco type groups are defined as follows: smokers (all participants that self-reported as smoker independently of cotinine results), exclusively cigarette smokers (those that only smoke cigarettes), exclusively dokha smokers (those that only smoke dokha, typically in the traditional midwakh pipe), exclusively shisha smokers (those that only smoke shisha), multiple smokers (those that smoke more than one type of tobacco product), nonsmokers (those that self-reported as nonsmokers, and were further validated by a cotinine negative result).

#### Mouthwash sample collection

Participants were given a 10 ml sample of pharmaceutical grade normal saline (0.9%) solution and asked to vigorously swish for 30 seconds and spit it out onto a new sterile tube. Samples were stored initially at 4 °C for no more than 48 h. Samples were then vortexed for 20 seconds, pipetted up and down 10 times, aliquoted into 1 ml cryotubes and stored at −80 °C until further processing. To confirm that the saline solution used for collection of mouthwash samples contained no detectable levels of DNA, identical DNA extraction methods to those used in the study (see below), were applied to two separate saline solutions samples alongside two mouthwash samples. Neither of the two saline solution samples yielded any DNA. Extracted DNA was viewed by gel electrophoresis and concentrations were quantified using the high sensitivity Qubit assay. Only mouthwash samples yielded measurable amounts of DNA (Supplementary Table S5).

#### Microbiome assay

Two 1 ml aliquots per sample were pooled for DNA extraction. Thawed samples were centrifuged at 6000 g for 3 min and then at 10000 g for 10 min in order to collect the cell pellet. DNA was extracted using the Mo BioPowerSoil PowerLyzer kit following manufacturer’s instructions (Mo Bio Laboratory Inc, California, USA). Genomic DNA was visualized on a gel and quantified using the Qubit HS kit (Thermo Fisher Scientific). Amplification of DNA from the V4 region of *16S rDNA* gene (515 F-5′GTGCCAGCMGCCGCGGTAA3′ - 806 R - 5′GGACTACHVGGGTWTCTAAT3′) was performed using specifically designed primers with Roche 454 FLK adaptor sequences and a 12 bp index (reverse primer only) added for posterior multiplexing. Amplification was carried out using the FastStart enzyme (Roche, IN). PCR products were visualized in an agarose gel, purified using Agencourt AMPure beads (Beckman Coulter Life Sciences, IN) and quantified using the Qubit BR kit (Thermo Fisher Scientific). Samples were then pooled for sequencing on an Illumina Miseq platform.

#### Quality control

Samples were sequenced in two batches. In addition to study samples, each batch contained three quality control samples, each in triplicate (shown in Supplementary Table S6) and a negative control (blank sample for DNA extraction and PCR amplification). Quality control samples showed good reliability, with the coefficient of variability ranging from 1.65–2.32% for the Shannon entropy and 1.02–7.21% for specific phyla relative abundances.

### Statistical analysis

#### Sequence data processing and taxonomic assignment

Sequences were de-multiplexed and trimmed using the split_libraries_fastq.py QIIME script with default parameters^53^. Only sequences that passed quality control filters (average base score quality per read .20, reads longer than 200 bp), were further processed. Taxonomical assignment was achieved using the pick_de_novo_otus.py workflow as implemented in QIIME^53^. Sequences were clustered into operational taxonomical units (OTU) using a 97% pairwise-identity cutoff, executing the UCLUST algorithm^54^. PyNAST^55^ and the Greengenes database were used for taxonomical assignment, followed by removal of chimeric sequences using ChimeraSlayer as implemented in the QIIME workflow^51^. Low count OTUs were filtered from the analyses if they were singletons and absent in more than 10% of the participants.

#### Estimating α-diversity

*β-diversity and taxa relative abundances*. Oral microbiome richness and diversity were estimated from a rarefied dataset (16738 sequence reads per sample), in order to eliminate possible biases introduced by differences of sampling effort. Estimation of richness (observed and Chao) and diversity (Shannon entropy and Simpson diversity index) were calculated using the vegan library in R^56^ (Supplementary Figure S3). To compare α-diversity between cases and controls we modeled richness and Shannon entropy in linear regression, adjusting for age sex and batch effects. Because linear regression assumes a normal distribution of the outcome, Shannon entropy was previously log transformed. We conducted permutational multivariate analysis of variance (PERMANOVA) of weighted (taxa relative abundance) and unweighted (absence/presence) Unifrac distance matrices to compare overall oral microbial composition between tobacco users and nonusers and by tobacco type^56^. Matrices were calculated implementing the Unifrac function in the Phyloseq library in R^57,58^. We then generated PCoA plots to visualize sample ordination using the first two principal coordinates. All PERMANOVA analyses were adjusted for age, sex, and batch effects and were performed using the Adonis function in the vegan R library^56^. We used DESeq2^59^ to explore for differential taxa abundances between smokers and nonsmokers for all tobacco categories as well as for the cotinine data. All statistical tests were two-sided. A p-value < 0.05 was considered of nominal statistical significance. In order to limit false detection of significance due to multiple comparisons, we adjusted for the False Discovery Rate (FDR)^60^. We determined a q-value < 0.10 as significant after adjustment. All analyses were conducted using R version 3.3.2^61^.

### Data availability

The datasets analyzed in this study are available in the Qiita database study ID - 11838.

## Electronic supplementary material

Supplementary Information
