# Supplementary material for: Types of tobacco consumption and the oral microbiome in the United Arab Emirates Healthy Future (UAEHFS) Pilot Study
Source: Sci Rep. 2018 Jul 27;8:11327. doi: 10.1038/s41598-018-29730-x (PMC6063860; doi:10.1038/s41598-018-29730-x)

Supplementary Information

Types of tobacco consumption and the oral microbiome in the United Arab Emirates Healthy Future (UAEHFS) Pilot Study.

Yvonne Vallès[1], Claire K. Inman[1], Brandilyn A. Peters[2], Raghib Ali[1], Laila Abdel Wareth[3], Abdishakur Abdulle[1], Habiba Alsafar[4,5], Fatme Al Anouti[6], Ayesha Al Dhaheri[7], Divya Galani[1], Muna Haji[1], Aisha Al Hamiz[1], Ayesha Al Hosani[1], Mohammed Al Houqani[8], Abdulla Al Junaibi[9], Marina Kazim[10], Tomas Kirchhoff[2], Wael Al Mahmeed[11], Fatma Al Maskari[12], Abdullah Alnaeemi[13], Naima Oumeziane[14], Ravichandran Ramasamy[15], Ann Marie Schmidt[15], Michael Weitzman[1,16,17], Eiman Al Zaabi[10], Scott Sherman[1,2], Richard B. Hayes[2,18] and Jiyoung Ahn[2,18*]

1. Public Health Research Center, New York University Abu Dhabi, Abu Dhabi, UAE;
2. Department of Population Health, New York University School of Medicine, New York, USA;
3. Pathology and Laboratory Medicine Institute, Cleveland Clinic, Abu Dhabi, UAE;
4. Center for Biotechnology, Khalifa University of Science and Technology, Abu Dhabi, UAE;
5. Biomedical Engineering Department, Khalifa University of Science and Technology, Abu Dhabi, UAE;
6. College of Natural and Health Sciences, Zayed University, Abu Dhabi, UAE;
7. Department of Nutrition, College of Food and Agriculture; UAE University, Al-Ain, UAE;
8. Department of Medicine, College of Medicine and Health Sciences, UAE University, Al-Ain, UAE;
9. Department of Pediatrics, Zayed Military Hospital, Abu Dhabi, UAE;
10. Department of Pathology, Sheikh Khalifa Medical City, Abu Dhabi, UAE;
11. Heart and Vascular Institue, Cleveland Clinic, Abu Dhabi, UAE;
12. Institute of Public Health, College of Medicine and Health Sciences, UAE University, Al-Ain, UAE.
13. Department of Cardiology, Zayed Military Hospital, Abu Dhabi, UAE;
14. Abu Dhabi Blood Bank, SEHA, Abu Dhabi, UAE;
15. Diabetes Research Program, Division of Endocrinology, Diabetes and Metabolism, Department of Medicine, New York University School of Medicine, New York, USA;
16. Department of Environmental Medicine, New York University School of Medicine, New York, USA;
17. Department of Pediatrics, New York University School of Medicine, New York, USA
18. NYU Perlmutter Cancer Center, New York, USA

*Corresponding author Jiyoung.Ahn@nyumc.org

Supplementary Table S1. Phyla distribution in the Emirati oral microbiome.

Phylum	Abundance %
Firmicutes	50.000
Bacteroidetes	21.684
Proteobacteria	15.752
Actinobacteria	6.731
Fusobacteria	4.713
Spirochaetes	0.656
SR1	0.258
Synergistetes	0.093
Tenericutes	0.092
Cyanobacteria	0.010
GN02	0.010
TM7	0.002
Chloroflexi	0.001

Figure S1. Principal Coordinate Analysis (PCoA) of the bacterial communities derived from Unifrac weighted distances and according to cotinine results. Overall microbial communities were significantly different between cotinine positive and cotinine negative participants (p=0.001). All cotinine negative participants were colored orange and all cotinine positive in blue. PCoA was based on the Unifrac weighted matrix at the OTU level.

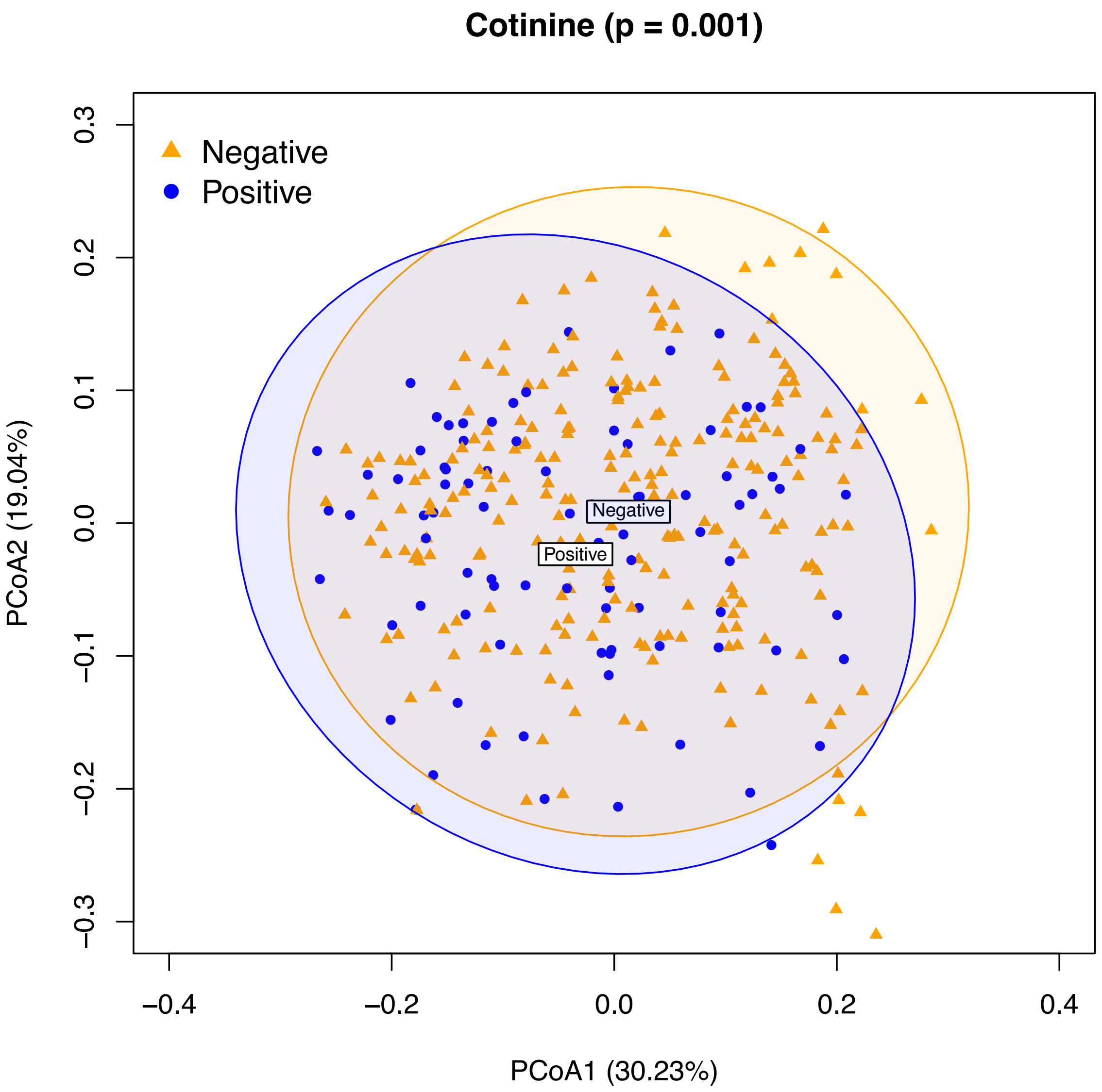

Figure S2. Principal Coordinate Analysis (PCoA) of the bacterial communities derived from Unifrac weighted distances and according to tobacco types. Overall microbial communities were not significantly different amongst the three tobacco types (p=0.2). PCoA was based on the Unifrac weighted matrix at the OTU level.

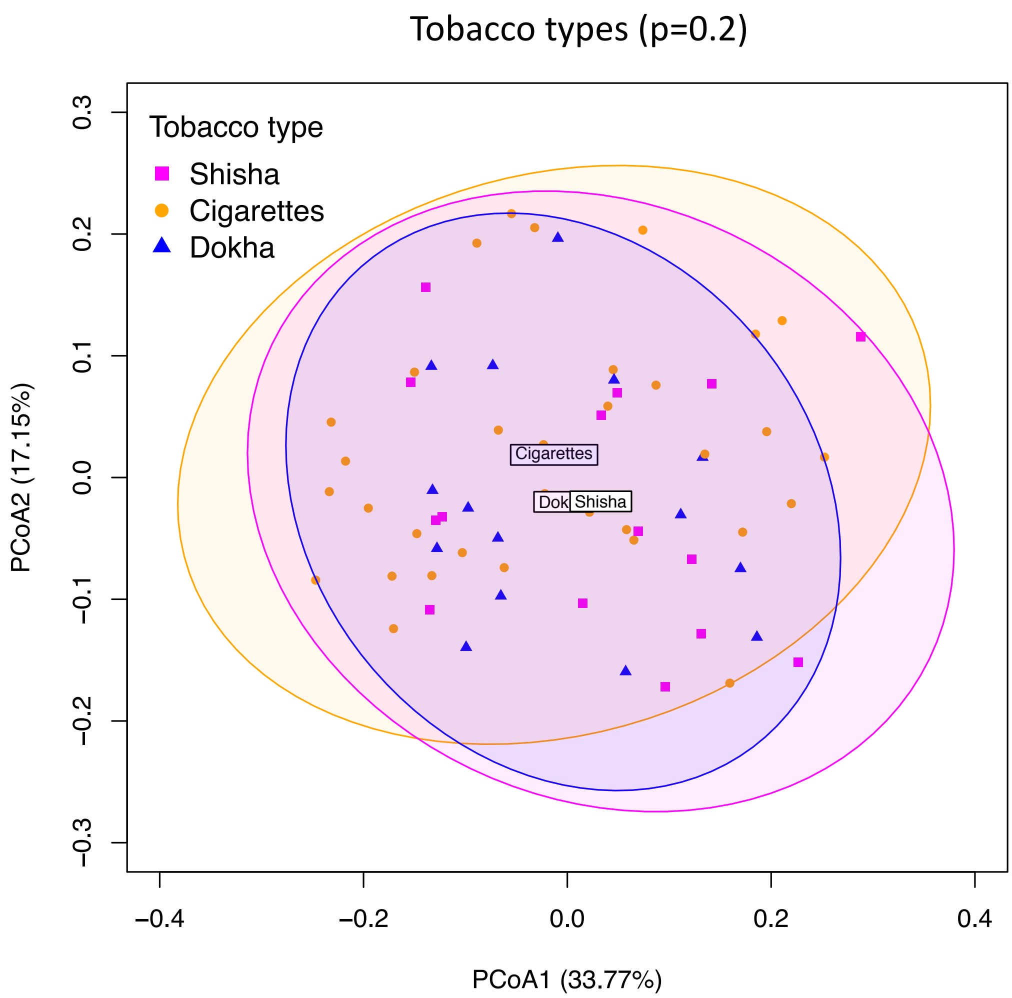

Supplementary Table S2. Differentially abundant taxa at all taxonomical levels in the contrasts between tobacco type smokers versus nonsmokers

Taxa[1]	Non Smokers	Cigarettes			Dokha			Shisha			Multiple		
	Mean[2]	Mean[2]	Log2FC(95CI %)	q[3]	Mean[2]	Log2FC(95CI %)	q[3]	Mean[2]	Log2FC(95CI %)	q[3]	Mean[2]	Log2FC(95CI %)	q[3]
**Phylum**													
Cyanobacteria	5.76	0.83	-1.38 (-2.36, -0.39)	1.00E-02	0.00	-1.40 (-2.32, -0.48)	4.00E-02	1.31	-1.16 (-2.08, -0.25)	8.00E-02	0.90	-1.30 (-2.29, -0.31)	3.00E-02
Fusobacteria	1733.44	1254.80	-0.48 (-0.73, -0.23)	0.00E+00	1788.84	0.08 (-0.26, 0.41)	8.50E-01	1770.45	0.02 (-0.32, 0.36)	1.00E+00	1739.36	0.06 (-0.17, 0.30)	6.40E-01
GN02	3.27	0.52	-2.03 (-2.83, -1.22)	0.00E+00	1.75	-0.86 (-1.74, 0.02)	2.50E-01	2.23	-0.83 (-1.72, 0.05)	2.80E-01	3.17	-0.35 (-1.10, 0.40)	5.90E-01
Proteobacteria	7106.72	3563.83	-1.08 (-1.44, -0.72)	0.00E+00	6666.25	-0.20 (-0.67, 0.27)	7.60E-01	6525.77	-0.26 (-0.74, 0.22)	9.10E-01	4984.18	-0.56 (-0.90, -0.22)	1.00E-02
Spirochaetes	157.39	337.85	1.07 (0.52, 1.61)	0.00E+00	236.53	0.68 (0.00, 1.36)	2.50E-01	122.97	-0.11 (-0.79, 0.58)	1.00E+00	273.62	0.92 (0.40, 1.43)	1.00E-02
SR1	81.79	26.15	-1.31 (-2.01, -0.60)	0.00E+00	84.05	-0.28 (-1.10, 0.54)	8.20E-01	28.36	-1.10 (-1.92, -0.27)	8.00E-02	73.22	-0.40 (-1.07, 0.27)	4.60E-01
Synergistetes	18.53	81.96	1.72 (1.09, 2.35)	0.00E+00	23.42	0.66 (-0.11, 1.43)	3.00E-01	20.68	0.31 (-0.46, 1.09)	9.30E-01	36.90	1.03 (0.43, 1.64)	1.00E-02
Tenericutes	23.83	43.11	0.68 (0.06, 1.31)	5.00E-02	29.92	0.43 (-0.32, 1.19)	5.60E-01	16.83	-0.35 (-1.11, 0.42)	9.30E-01	30.30	0.47 (-0.12, 1.06)	3.10E-01
**Phylim;Class**													
Bacteroidetes;Flavobacteriia	301.77	136.35	-1.02 (-1.38, -0.66)	0.00E+00	211.98	-0.42 (-0.88, 0.04)	2.13E-01	333.38	0.07 (-0.40, 0.54)	9.93E-01	226.76	-0.34 (-0.68, 0.00)	1.27E-01
Cyanobacteria;Chloroplast	6.91	1.13	-1.05 (-1.82, -0.29)	2.00E-02	0.00	-1.06 (-1.75, -0.38)	4.88E-02	1.36	-0.94 (-1.63, -0.26)	8.98E-02	0.98	-1.13 (-1.90, -0.35)	1.66E-02
Firmicutes;Erysipelotrichi	73.74	57.68	-0.43 (-0.84, -0.02)	8.00E-02	56.90	-0.48 (-0.99, 0.04)	2.13E-01	83.20	0.00 (-0.52, 0.52)	9.93E-01	71.43	-0.22 (-0.60, 0.17)	3.84E-01
Fusobacteria;Fusobacteriia	1779.77	1359.71	-0.41 (-0.63, -0.18)	0.00E+00	1678.42	-0.07 (-0.37, 0.23)	7.30E-01	1766.27	-0.02 (-0.33, 0.29)	9.93E-01	1776.30	0.03 (-0.18, 0.25)	8.05E-01
GN02;BD1-5	3.95	0.56	-1.81 (-2.53, -1.10)	0.00E+00	1.95	-0.93 (-1.67, -0.19)	1.17E-01	1.90	-0.98 (-1.72, -0.25)	8.98E-02	3.62	-0.45 (-1.14, 0.23)	3.39E-01
Proteobacteria;Betaproteobacteria	2896.63	1105.40	-1.43 (-1.88, -0.98)	0.00E+00	2352.09	-0.51 (-1.07, 0.05)	2.13E-01	2331.47	-0.50 (-1.07, 0.06)	4.02E-01	1841.22	-0.80 (-1.23, -0.37)	2.53E-03
Proteobacteria;Deltaproteobacteria	0.72	1.06	0.42 (-0.34, 1.19)	3.20E-01	1.26	0.54 (-0.20, 1.28)	3.43E-01	0.54	0.21 (-0.53, 0.95)	9.93E-01	1.89	0.94 (0.19, 1.69)	4.05E-02
Proteobacteria;Epsilonproteobacteria	149.83	164.23	0.23 (-0.01, 0.48)	1.20E-01	172.36	0.38 (0.06, 0.71)	1.17E-01	147.77	0.09 (-0.24, 0.42)	9.93E-01	183.44	0.46 (0.23, 0.69)	2.12E-03
Proteobacteria;Gammaproteobacteria	4361.81	2647.16	-0.79 (-1.15, -0.43)	0.00E+00	4002.36	-0.16 (-0.62, 0.31)	6.75E-01	3996.98	-0.25 (-0.72, 0.22)	9.93E-01	3034.78	-0.49 (-0.83, -0.15)	1.66E-02
Spirochaetes;Spirochaetes	175.62	351.83	0.91 (0.39, 1.44)	0.00E+00	223.11	0.41 (-0.21, 1.04)	3.96E-01	129.73	-0.12 (-0.75, 0.51)	9.93E-01	296.30	0.77 (0.27, 1.27)	1.36E-02
Synergistetes;Synergistia	20.17	71.63	1.43 (0.84, 2.02)	0.00E+00	21.31	0.41 (-0.26, 1.09)	4.15E-01	21.28	0.29 (-0.39, 0.96)	9.93E-01	39.86	0.92 (0.36, 1.48)	8.93E-03
Tenericutes;Mollicutes	25.66	47.98	0.69 (0.10, 1.27)	5.00E-02	26.41	0.16 (-0.51, 0.83)	7.30E-01	15.66	-0.34 (-1.02, 0.33)	9.93E-01	32.13	0.36 (-0.20, 0.92)	3.39E-01
**Phylum;Class;Order**													
Actinobacteria;Actinobacteria;Bifidobacteriales	47.42	65.70	0.71 (0.09, 1.32)	4.35E-02	98.49	0.98 (0.27, 1.69)	4.35E-02	39.89	0.18 (-0.54, 0.89)	9.96E-01	52.23	0.41 (-0.18, 0.99)	3.14E-01
Actinobacteria;Coriobacteriia;Coriobacteriales	163.68	231.28	0.45 (0.00, 0.91)	8.59E-02	232.02	0.40 (-0.17, 0.97)	3.06E-01	194.95	0.21 (-0.36, 0.79)	9.96E-01	139.40	-0.25 (-0.68, 0.18)	4.14E-01
Bacteroidetes;Flavobacteriia;Flavobacteriales	287.70	136.29	-0.92 (-1.28, -0.57)	4.68E-06	207.39	-0.36 (-0.82, 0.10)	2.51E-01	313.24	0.12 (-0.35, 0.59)	9.96E-01	211.25	-0.36 (-0.69, -0.02)	9.24E-02
Cyanobacteria;Chloroplast;Streptophyta	6.80	1.00	-1.17 (-1.99, -0.35)	1.26E-02	0.00	-1.20 (-1.95, -0.45)	2.03E-02	1.18	-1.06 (-1.80, -0.32)	1.29E-01	0.81	-1.28 (-2.11, -0.45)	1.53E-02
Firmicutes;Bacilli;Gemellales	1323.12	847.98	-0.63 (-0.98, -0.29)	1.10E-03	788.14	-0.73 (-1.17, -0.28)	2.03E-02	1353.85	0.01 (-0.45, 0.46)	9.96E-01	1024.84	-0.41 (-0.74, -0.08)	4.28E-02
Firmicutes;Clostridia;Clostridiales	4015.00	4695.19	0.25 (-0.02, 0.52)	9.51E-02	5282.44	0.43 (0.07, 0.79)	7.62E-02	4135.83	0.05 (-0.32, 0.41)	9.96E-01	3923.02	-0.04 (-0.29, 0.22)	8.48E-01
Fusobacteria;Fusobacteriia;Fusobacteriales	1726.06	1391.18	-0.33 (-0.56, -0.09)	1.39E-02	1651.92	-0.02 (-0.34, 0.29)	9.58E-01	1797.20	0.05 (-0.27, 0.37)	9.96E-01	1681.21	0.02 (-0.20, 0.24)	8.88E-01
Proteobacteria;Betaproteobacteria;Burkholderiales	199.61	72.60	-1.25 (-1.84, -0.65)	1.88E-04	80.82	-1.00 (-1.69, -0.31)	3.96E-02	129.94	-0.59 (-1.28, 0.11)	6.25E-01	179.53	-0.21 (-0.78, 0.35)	6.07E-01
Proteobacteria;Betaproteobacteria;Neisseriales	2475.77	989.16	-1.37 (-1.82, -0.91)	1.08E-07	2094.47	-0.47 (-1.04, 0.10)	2.51E-01	2112.25	-0.41 (-0.98, 0.17)	8.40E-01	1538.68	-0.84 (-1.27, -0.41)	3.43E-03
Proteobacteria;Deltaproteobacteria;Desulfobacterales	0.75	1.14	0.43 (-0.38, 1.24)	3.53E-01	1.24	0.52 (-0.27, 1.32)	3.28E-01	0.55	0.18 (-0.62, 0.97)	9.96E-01	1.83	0.92 (0.12, 1.71)	6.51E-02
Proteobacteria;Epsilonproteobacteria;Campylobacterales	146.83	173.22	0.34 (0.08, 0.60)	2.03E-02	170.12	0.43 (0.08, 0.77)	7.38E-02	148.23	0.13 (-0.22, 0.48)	9.96E-01	176.42	0.45 (0.21, 0.69)	3.51E-03
Proteobacteria;Gammaproteobacteria;Cardiobacteriales	21.40	10.34	-0.78 (-1.28, -0.27)	7.48E-03	17.11	-0.18 (-0.80, 0.44)	6.71E-01	30.25	0.29 (-0.33, 0.91)	9.96E-01	12.56	-0.40 (-0.88, 0.08)	2.06E-01
Proteobacteria;Gammaproteobacteria;Pasteurellales	4044.63	2638.41	-0.67 (-1.02, -0.32)	6.37E-04	3700.93	-0.15 (-0.60, 0.31)	6.60E-01	3725.33	-0.20 (-0.66, 0.26)	9.96E-01	2759.22	-0.51 (-0.84, -0.18)	1.53E-02
Proteobacteria;Gammaproteobacteria;Pseudomonadales	11.46	2.54	-0.80 (-1.63, 0.02)	8.91E-02	15.38	-0.17 (-0.95, 0.60)	7.50E-01	7.53	-0.36 (-1.13, 0.42)	9.96E-01	13.10	-0.18 (-1.01, 0.65)	7.80E-01
Spirochaetes;Spirochaetes;Spirochaetales	179.27	405.08	1.05 (0.50, 1.60)	6.37E-04	242.55	0.48 (-0.17, 1.14)	2.86E-01	134.04	-0.12 (-0.78, 0.54)	9.96E-01	290.12	0.74 (0.22, 1.26)	1.94E-02
Synergistetes;Synergistia;Synergistales	20.45	78.45	1.52 (0.91, 2.13)	8.43E-06	22.93	0.44 (-0.26, 1.15)	3.40E-01	23.29	0.33 (-0.38, 1.04)	9.96E-01	37.16	0.84 (0.26, 1.43)	1.89E-02
Tenericutes;Mollicutes;Mycoplasmatales	15.82	47.94	1.48 (0.82, 2.14)	6.64E-05	20.10	0.74 (0.00, 1.48)	1.77E-01	11.77	0.22 (-0.52, 0.96)	9.96E-01	22.84	0.94 (0.31, 1.57)	1.69E-02
Tenericutes;Mollicutes;RF39	10.44	5.16	-0.91 (-1.64, -0.17)	2.98E-02	7.61	-0.63 (-1.40, 0.15)	2.51E-01	4.51	-0.87 (-1.65, -0.09)	2.45E-01	8.27	-0.55 (-1.26, 0.16)	2.46E-01
Tenericutes;RF3;ML615J-28	0.34	1.23	0.78 (-0.04, 1.60)	9.12E-02	1.03	0.67 (-0.08, 1.42)	2.49E-01	1.91	0.83 (0.08, 1.57)	2.45E-01	0.97	0.74 (-0.09, 1.57)	1.87E-01

[1] Only those taxa that have a significantly differential abundance with q<0.1 and a cook's distance <10 in at least one contrast are shown.

[2] Mean values refer to mean normalized counts of taxa according to each group.

[3] FDR adjusted p value. FDR adjustment was implemented at each level independently (i.e. class, order)

Supplementary Table S2 continued.

Taxa[1]	Non smokers	Cigarettes			Dokha			Shisha			Multiple		
	Mean[2]	Mean[2]	Log2FC(CI 95%)	q[3]	Mean[2]	Log2FC(CI 95%)	q[3]	Mean[2]	Log2FC(CI 95%)	q[3]	Mean[2]	Log2FC(CI 95%)	q[3]
Phylum;Class;Order;Family													
Actinobacteria;Actinobacteria;Bifidobacteriales;Bifidobacteriaceae	50.15	64.62	0.72 (0.08, 1.36)	5.70E-02	96.45	0.97 (0.22, 1.72)	9.10E-02	37.92	0.11 (-0.65, 0.86)	9.94E-01	51.98	0.41 (-0.20, 1.02)	4.24E-01
Actinobacteria;Coriobacteriia;Coriobacteriales;Coriobacteriaceae	159.83	258.13	0.65 (0.19, 1.12)	1.68E-02	226.07	0.45 (-0.13, 1.04)	3.10E-01	176.91	0.17 (-0.43, 0.76)	9.94E-01	141.81	-0.14 (-0.58, 0.30)	7.46E-01
Bacteroidetes;Bacteroidia;Bacteroidales;[Paraprevotellaceae]	739.19	1087.74	0.74 (0.34, 1.14)	1.12E-03	792.32	0.20 (-0.31, 0.72)	6.24E-01	866.50	0.44 (-0.08, 0.97)	9.94E-01	817.56	0.17 (-0.21, 0.55)	6.39E-01
Bacteroidetes;Bacteroidia;Bacteroidales;Porphyromonadaceae	942.40	747.31	-0.43 (-0.79, -0.07)	4.43E-02	639.25	-0.75 (-1.22, -0.28)	2.72E-02	866.19	-0.30 (-0.78, 0.18)	9.94E-01	966.10	-0.21 (-0.55, 0.13)	4.58E-01
Bacteroidetes;Flavobacteriia;Flavobacteriales;[Weeksellaceae]	135.15	54.21	-1.05 (-1.48, -0.61)	2.88E-05	55.77	-0.94 (-1.49, -0.39)	2.72E-02	156.61	0.06 (-0.49, 0.62)	9.94E-01	67.73	-0.78 (-1.18, -0.37)	3.73E-03
Bacteroidetes;Flavobacteriia;Flavobacteriales;Flavobacteriaceae	155.47	83.60	-0.79 (-1.19, -0.40)	4.05E-04	149.48	-0.01 (-0.52, 0.50)	9.70E-01	165.30	0.14 (-0.37, 0.65)	9.94E-01	138.84	-0.11 (-0.47, 0.26)	7.46E-01
Firmicutes;Bacilli;Gemellales;Gemellaceae	1338.32	849.07	-0.65 (-1.01, -0.29)	1.30E-03	778.55	-0.74 (-1.20, -0.27)	2.72E-02	1387.88	0.01 (-0.47, 0.48)	9.94E-01	991.61	-0.44 (-0.78, -0.10)	4.63E-02
Firmicutes;Bacilli;Lactobacillales;Aerococcaceae	107.62	43.06	-1.26 (-1.92, -0.60)	8.41E-04	58.85	-0.92 (-1.69, -0.15)	1.10E-01	56.63	-0.82 (-1.59, -0.05)	9.94E-01	103.10	-0.35 (-0.98, 0.27)	5.24E-01
Firmicutes;Clostridia;Clostridiales;[Mogibacteriaceae]	109.65	154.58	0.47 (0.09, 0.84)	3.88E-02	119.39	0.14 (-0.35, 0.63)	6.96E-01	108.82	-0.02 (-0.52, 0.48)	9.94E-01	130.13	0.26 (-0.09, 0.62)	3.78E-01
Firmicutes;Clostridia;Clostridiales;Veillonellaceae	3187.29	4162.30	0.41 (0.07, 0.74)	3.88E-02	4248.81	0.50 (0.06, 0.94)	1.36E-01	3176.71	0.04 (-0.40, 0.49)	9.94E-01	3076.86	0.03 (-0.29, 0.34)	9.30E-01
Fusobacteria;Fusobacteriia;Fusobacteriales;Fusobacteriaceae	1010.16	805.57	-0.33 (-0.60, -0.06)	3.88E-02	799.50	-0.29 (-0.65, 0.07)	3.10E-01	888.86	-0.17 (-0.54, 0.19)	9.94E-01	946.30	-0.04 (-0.30, 0.21)	8.56E-01
Proteobacteria;Betaproteobacteria;Burkholderiales;Burkholderiaceae	214.03	72.21	-1.35 (-1.98, -0.72)	2.09E-04	78.39	-1.09 (-1.83, -0.34)	4.25E-02	128.63	-0.65 (-1.40, 0.10)	9.94E-01	173.11	-0.29 (-0.89, 0.30)	6.27E-01
Proteobacteria;Betaproteobacteria;Burkholderiales;Comamonadaceae	5.53	0.44	-1.63 (-2.43, -0.83)	4.05E-04	1.77	-0.48 (-1.33, 0.37)	4.82E-01	4.53	-0.15 (-1.00, 0.70)	9.94E-01	1.03	-1.12 (-1.89, -0.35)	2.58E-02
Proteobacteria;Betaproteobacteria;Neisseriales;Neisseriaceae	2527.04	979.39	-1.42 (-1.89, -0.95)	1.22E-07	2078.77	-0.47 (-1.06, 0.12)	3.10E-01	2142.55	-0.41 (-1.01, 0.19)	9.94E-01	1484.77	-0.89 (-1.33, -0.44)	3.60E-03
Proteobacteria;Deltaproteobacteria;Desulfobacterales;Desulfobulbaceae	0.73	1.21	0.52 (-0.35, 1.38)	3.49E-01	1.42	0.68 (-0.19, 1.56)	3.10E-01	0.62	0.23 (-0.64, 1.10)	9.94E-01	1.74	1.00 (0.16, 1.85)	8.08E-02
Proteobacteria;Epsilonproteobacteria;Campylobacterales;Campylobacteraceae	147.70	177.20	0.37 (0.10, 0.64)	1.87E-02	167.89	0.43 (0.07, 0.79)	1.10E-01	146.48	0.12 (-0.24, 0.49)	9.94E-01	170.57	0.44 (0.19, 0.69)	7.32E-03
Proteobacteria;Gammaproteobacteria;Cardiobacteriales;Cardiobacteriaceae	22.70	10.31	-0.86 (-1.40, -0.33)	4.69E-03	16.84	-0.26 (-0.92, 0.39)	6.24E-01	30.69	0.27 (-0.39, 0.93)	9.94E-01	13.11	-0.39 (-0.90, 0.11)	3.64E-01
Proteobacteria;Gammaproteobacteria;Pasteurellales;Pasteurellaceae	4104.09	2684.82	-0.68 (-1.05, -0.32)	9.91E-04	3700.57	-0.14 (-0.62, 0.33)	6.96E-01	3853.95	-0.19 (-0.67, 0.29)	9.94E-01	2790.37	-0.49 (-0.83, -0.15)	2.58E-02
Spirochaetes;Spirochaetes;Spirochaetales;Spirochaetaceae	169.21	390.20	1.12 (0.56, 1.67)	4.05E-04	230.82	0.53 (-0.15, 1.21)	3.10E-01	145.50	0.00 (-0.69, 0.68)	9.94E-01	285.94	0.83 (0.30, 1.35)	1.34E-02
Synergistetes;Synergistia;Synergistales;Dethiosulfovibrionaceae	19.56	79.73	1.61 (0.98, 2.23)	1.06E-05	22.32	0.51 (-0.23, 1.25)	3.35E-01	26.27	0.46 (-0.29, 1.21)	9.94E-01	39.55	0.99 (0.40, 1.59)	9.93E-03
Tenericutes;Mollicutes;Mycoplasmatales;Mycoplasmataceae	15.17	45.64	1.51 (0.83, 2.19)	1.45E-04	19.53	0.81 (0.03, 1.59)	1.96E-01	13.08	0.31 (-0.48, 1.09)	9.94E-01	23.48	1.07 (0.42, 1.72)	9.93E-03
Phylum;Class;Order;Family;Genus													
Actinobacteria;Actinobacteria;Bifidobacteriales;Bifidobacteriaceae;Bifidobacterium	6.51	13.16	0.84 (0.03, 1.65)	1.00E-01	57.14	1.88 (1.01, 2.74)	1.17E-03	8.09	0.49 (-0.37, 1.36)	8.95E-01	8.81	0.49 (-0.29, 1.27)	4.45E-01
Actinobacteria;Coriobacteriia;Coriobacteriales;Coriobacteriaceae;Atopobium	142.80	221.05	0.59 (0.14, 1.05)	4.00E-02	181.90	0.31 (-0.27, 0.89)	6.74E-01	144.84	0.05 (-0.54, 0.64)	9.70E-01	117.81	-0.26 (-0.69, 0.17)	4.48E-01
Bacteroidetes;Bacteroidia;Bacteroidales;[Paraprevotellaceae];[Prevotella]	732.67	986.83	0.58 (0.18, 0.97)	2.00E-02	715.75	0.06 (-0.45, 0.58)	8.96E-01	856.09	0.41 (-0.11, 0.94)	8.95E-01	717.10	-0.01 (-0.38, 0.37)	9.64E-01
Bacteroidetes;Bacteroidia;Bacteroidales;Porphyromonadaceae;Paludibacter	23.68	38.59	0.68 (0.12, 1.24)	5.00E-02	22.67	0.19 (-0.50, 0.88)	7.83E-01	13.34	-0.37 (-1.07, 0.33)	8.95E-01	30.94	0.64 (0.11, 1.17)	8.04E-02
Bacteroidetes;Bacteroidia;Bacteroidales;Porphyromonadaceae;Porphyromonas	913.25	618.01	-0.67 (-1.07, -0.26)	1.00E-02	552.77	-0.93 (-1.45, -0.40)	1.44E-02	701.09	-0.53 (-1.06, 0.00)	7.36E-01	841.62	-0.39 (-0.77, -0.01)	1.58E-01
Bacteroidetes;Flavobacteriia;Flavobacteriales;Flavobacteriaceae;Capnocytophaga	157.25	75.70	-0.95 (-1.35, -0.54)	0.00E+00	141.59	-0.11 (-0.63, 0.41)	8.29E-01	171.86	0.15 (-0.38, 0.68)	9.70E-01	130.23	-0.21 (-0.59, 0.17)	4.91E-01
Firmicutes;Bacilli;Gemellales;Gemellaceae;Gemella	23.11	16.75	-0.57 (-1.03, -0.12)	4.00E-02	18.68	-0.42 (-1.00, 0.15)	4.79E-01	29.34	0.19 (-0.39, 0.77)	9.70E-01	14.68	-0.73 (-1.16, -0.31)	1.68E-02
Firmicutes;Bacilli;Lactobacillales;Enterococcaceae;Vagococcus	1.25	0.91	-0.36 (-0.98, 0.25)	3.70E-01	1.27	-0.19 (-0.92, 0.54)	7.83E-01	1.25	-0.09 (-0.82, 0.65)	9.70E-01	0.71	-0.73 (-1.31, -0.15)	7.20E-02
Firmicutes;Clostridia;Clostridiales;Peptostreptococcaceae;Peptostreptococcus	95.48	54.49	-0.77 (-1.29, -0.24)	2.00E-02	53.88	-0.79 (-1.44, -0.14)	2.04E-01	95.87	-0.14 (-0.80, 0.52)	9.70E-01	74.96	-0.44 (-0.94, 0.05)	2.29E-01
Firmicutes;Clostridia;Clostridiales;Veillonellaceae;Dialister	54.07	63.92	0.45 (0.05, 0.84)	8.00E-02	47.16	0.10 (-0.42, 0.61)	8.29E-01	53.78	0.17 (-0.35, 0.69)	9.70E-01	46.95	0.08 (-0.30, 0.45)	8.29E-01
Firmicutes;Clostridia;Clostridiales;Veillonellaceae;Megasphaera	156.45	266.63	0.70 (0.19, 1.20)	3.00E-02	228.18	0.49 (-0.14, 1.12)	4.79E-01	160.45	0.05 (-0.59, 0.69)	9.70E-01	150.16	-0.05 (-0.53, 0.42)	8.60E-01
Fusobacteria;Fusobacteriia;Fusobacteriales;Fusobacteriaceae;Fusobacterium	989.40	743.50	-0.43 (-0.70, -0.16)	1.00E-02	747.78	-0.37 (-0.73, -0.01)	3.52E-01	822.11	-0.27 (-0.63, 0.10)	8.95E-01	890.29	-0.13 (-0.38, 0.12)	5.07E-01
Fusobacteria;Fusobacteriia;Fusobacteriales;Leptotrichiaceae;Leptotrichia	629.22	505.20	-0.35 (-0.66, -0.03)	9.00E-02	586.31	0.00 (-0.42, 0.42)	9.89E-01	777.87	0.29 (-0.14, 0.71)	8.95E-01	596.20	0.04 (-0.26, 0.33)	8.60E-01
Proteobacteria;Betaproteobacteria;Burkholderiales;Burkholderiaceae;Lautropia	232.27	68.79	-1.45 (-2.09, -0.81)	0.00E+00	80.40	-1.12 (-1.88, -0.37)	5.08E-02	130.76	-0.69 (-1.45, 0.07)	7.36E-01	161.85	-0.44 (-1.05, 0.17)	3.58E-01
Proteobacteria;Betaproteobacteria;Neisseriales;Neisseriaceae;Eikenella	27.43	11.01	-1.16 (-1.67, -0.66)	0.00E+00	26.74	-0.07 (-0.70, 0.55)	8.96E-01	54.70	0.78 (0.14, 1.41)	7.36E-01	15.48	-0.75 (-1.22, -0.27)	1.80E-02
Proteobacteria;Betaproteobacteria;Neisseriales;Neisseriaceae;Neisseria	2527.32	896.43	-1.51 (-2.03, -0.99)	0.00E+00	2053.66	-0.49 (-1.14, 0.15)	4.79E-01	2184.49	-0.43 (-1.09, 0.22)	8.95E-01	1326.65	-1.02 (-1.51, -0.53)	2.59E-03
Proteobacteria;Epsilonproteobacteria;Campylobacterales;Campylobacteraceae; Campylobacter	142.45	158.50	0.27 (0.02, 0.52)	9.00E-02	143.80	0.24 (-0.09, 0.57)	4.79E-01	141.67	0.11 (-0.23, 0.45)	9.70E-01	156.84	0.36 (0.13, 0.59)	1.80E-02
Proteobacteria;Gammaproteobacteria;Cardiobacteriales;Cardiobacteriaceae; Cardiobacterium	23.01	9.58	-1.00 (-1.53, -0.46)	0.00E+00	14.45	-0.46 (-1.12, 0.20)	4.79E-01	30.17	0.21 (-0.46, 0.87)	9.70E-01	11.18	-0.62 (-1.12, -0.12)	7.52E-02
Proteobacteria;Gammaproteobacteria;Pasteurellales;Pasteurellaceae;Actinobacillus	31.13	17.96	-0.79 (-1.41, -0.17)	4.00E-02	11.02	-1.14 (-1.88, -0.40)	4.83E-02	88.26	0.84 (0.09, 1.58)	7.36E-01	16.37	-0.90 (-1.49, -0.31)	1.98E-02
Proteobacteria;Gammaproteobacteria;Pasteurellales;Pasteurellaceae;Actinobacillus	31.13	17.96	-0.79 (-1.41, -0.17)	4.00E-02	11.02	-1.14 (-1.88, -0.40)	4.83E-02	88.26	0.84 (0.09, 1.58)	7.36E-01	16.37	-0.90 (-1.49, -0.31)	1.98E-02
Proteobacteria;Gammaproteobacteria;Pasteurellales;Pasteurellaceae;Aggregatibacter	353.06	196.38	-0.77 (-1.25, -0.29)	1.00E-02	257.84	-0.42 (-1.03, 0.18)	4.79E-01	494.65	0.37 (-0.24, 0.98)	8.95E-01	255.76	-0.46 (-0.91, -0.01)	1.58E-01
Proteobacteria;Gammaproteobacteria;Pasteurellales;Pasteurellaceae;Haemophilus	3786.72	2365.12	-0.77 (-1.16, -0.38)	0.00E+00	3404.50	-0.13 (-0.64, 0.37)	7.83E-01	3396.53	-0.29 (-0.81, 0.22)	8.95E-01	2333.62	-0.62 (-0.99, -0.26)	1.68E-02
Proteobacteria;Gammaproteobacteria;Pseudomonadales;Moraxellaceae; Enhydrobacter	2.18	2.25	0.21 (-0.71, 1.13)	8.10E-01	1.47	0.13 (-0.75, 1.01)	8.85E-01	1.91	0.22 (-0.65, 1.10)	9.70E-01	9.95	1.15 (0.23, 2.06)	7.20E-02
Spirochaetes;Spirochaetes;Spirochaetales;Spirochaetaceae;Treponema	161.93	346.73	1.08 (0.53, 1.63)	0.00E+00	201.42	0.43 (-0.24, 1.11)	5.36E-01	131.28	-0.06 (-0.75, 0.62)	9.70E-01	264.57	0.81 (0.29, 1.33)	1.80E-02
Synergistetes;Synergistia;Synergistales;Dethiosulfovibrionaceae;TG5	18.16	64.52	1.50 (0.89, 2.12)	0.00E+00	19.19	0.41 (-0.33, 1.15)	6.56E-01	23.06	0.40 (-0.35, 1.14)	8.95E-01	34.89	0.97 (0.38, 1.55)	1.68E-02
Tenericutes;Mollicutes;Mycoplasmatales;Mycoplasmataceae;Mycoplasma	14.78	40.78	1.41 (0.73, 2.09)	0.00E+00	15.67	0.55 (-0.24, 1.34)	4.79E-01	11.52	0.14 (-0.66, 0.93)	9.70E-01	21.21	0.93 (0.28, 1.57)	3.31E-02

[1] Only those taxa that have a significantly differential abundance with q<0.1 and a cook's distance <10 in at least one contrast are shown.
[2] Mean values refer to mean normalized counts of taxa according to each group.
[3] FDR adjusted p value. FDR adjustment was implemented at each level independently (i.e. class, order)

Supplementary Table S3. Differentially abundant taxa at all taxonomical levels between tobacco types.

Taxa[1]	Cigarettes	Dokha	Shisha	Cigarettes vs. Dokha		Cigarettes vs. Shisha		Dokha vs. Shisha	
	Mean[2]	Mean[2]	Mean[2]	log2FC (CI95%)	q[3]	log2FC (CI95%)	q[3]	log2FC (CI95%)	q[3]
Phylum									
Fusobacteria	1254.8	1788.84	1770.45	-0.46 (-0.90, -0.03)	2.04E-01	-0.48 (-0.90, -0.06)	9.38E-02	-0.02 (-0.51, 0.48)	9.59E-01
Proteobacteria	3563.83	6666.25	6525.77	-0.80 (-1.41, -0.20)	1.04E-01	-0.77 (-1.37, -0.18)	6.05E-02	0.03 (-0.65, 0.71)	9.59E-01
Spirochaetes	337.85	236.53	122.97	0.61 (-0.16, 1.38)	4.50E-01	1.25 (0.48, 2.02)	1.54E-02	0.65 (-0.21, 1.50)	9.59E-01
Class									
Bacteroidetes;Flavobacteriia	136.35	211.98	333.38	-0.52 (-1.05, 0.00)	1.94E-01	-1.11 (-1.63, -0.60)	5.13E-04	-0.59 (-1.18, 0.00)	5.02E-01
Proteobacteria; Betaproteobacteria	1105.4	2352.09	2331.47	-0.84 (-1.59, -0.09)	1.89E-01	-0.90 (-1.66, -0.14)	7.85E-02	-0.06 (-0.88, 0.76)	9.80E-01
Spirochaetes; Spirochaetes	351.83	223.11	129.73	0.71 (-0.02, 1.43)	1.94E-01	1.21 (0.47, 1.94)	1.26E-02	0.50 (-0.30, 1.30)	8.73E-01
Synergistetes;Synergistia	71.63	21.31	21.28	1.07 (0.26, 1.88)	1.89E-01	1.18 (0.36, 2.00)	3.28E-02	0.11 (-0.76, 0.98)	9.80E-01
Tenericutes;Mollicutes	47.98	26.41	15.66	0.56 (-0.24, 1.37)	3.74E-01	0.98 (0.17, 1.80)	7.85E-02	0.42 (-0.45, 1.28)	8.99E-01
Order									
Bacteroidetes;Flavobacteriia;Flavobacteriales	136.29	207.39	313.24	-0.48 (-0.99, 0.04)	3.93E-01	-0.99 (-1.50, -0.49)	2.97E-03	-0.51 (-1.10, 0.07)	6.93E-01
Firmicutes;Bacilli;Gemellales	847.98	788.14	1353.85	0.28 (-0.28, 0.83)	7.45E-01	-0.70 (-1.25, -0.15)	6.26E-02	-0.98 (-1.61, -0.35)	5.68E-02
Spirochaetes;Spirochaetes;Spirochaetales	405.08	242.55	134.04	0.70 (-0.08, 1.47)	3.93E-01	1.33 (0.55, 2.11)	1.04E-02	0.64 (-0.21, 1.49)	7.15E-01
Synergistetes;Synergistia;Synergistales	78.45	22.93	23.29	1.07 (0.22, 1.93)	3.45E-01	1.25 (0.39, 2.12)	3.82E-02	0.18 (-0.74, 1.10)	9.70E-01
Tenericutes;Mollicutes;Mycoplasmatales	47.94	20.1	11.77	0.73 (-0.17, 1.62)	3.96E-01	1.25 (0.35, 2.16)	4.16E-02	0.53 (-0.42, 1.48)	7.63E-01
Family									
Bacteroidetes;Flavobacteriia;Flavobacteriales;[Weeksellaceae]	54.21	55.77	156.61	0.03 (-0.58, 0.65)	9.46E-01	-1.09 (-1.69, -0.49)	1.49E-02	-1.12 (-1.81, -0.43)	2.79E-02
Bacteroidetes;Flavobacteriia;Flavobacteriales; Flavobacteriaceae	83.6	149.48	165.3	-0.70 (-1.28, -0.11)	2.64E-01	-0.86 (-1.43, -0.29)	3.15E-02	-0.16 (-0.81, 0.49)	8.05E-01
Firmicutes;Bacilli;Gemellales; Gemellaceae	849.07	778.55	1387.88	0.34 (-0.22, 0.89)	5.47E-01	-0.75 (-1.30, -0.21)	4.64E-02	-1.09 (-1.72, -0.46)	2.63E-02
Proteobacteria; Betaproteobacteria; BurkholderialesComamonadaceae	0.44	1.77	4.53	-0.74 (-1.75, 0.27)	5.47E-01	-1.65 (-2.68, -0.62)	2.14E-02	-0.91 (-1.90, 0.08)	4.75E-01
Spirochaetes;Spirochaetes;Spirochaetales;Spirochaetaceae	390.2	230.82	145.5	0.79 (-0.01, 1.58)	4.02E-01	1.33 (0.54, 2.11)	1.88E-02	0.54 (-0.33, 1.41)	6.31E-01
Synergistetes;Synergistia;Synergistales;Dethiosulfovibrionaceae	79.73	22.32	26.27	1.05 (0.16, 1.93)	2.64E-01	1.22 (0.33, 2.11)	4.64E-02	0.17 (-0.78, 1.13)	8.55E-01
Tenericutes;Mollicutes;Mycoplasmatales;Mycoplasmataceae	45.64	19.53	13.08	0.69 (-0.23, 1.61)	5.47E-01	1.20 (0.27, 2.13)	6.53E-02	0.51 (-0.48, 1.50)	6.76E-01
Genus									
Bacteroidetes;Bacteroidia;Bacteroidales;Porphyromonadaceae;Paludibacter	38.59	22.67	13.34	0.69 (-0.18, 1.57)	6.31E-01	1.24 (0.36, 2.12)	5.81E-02	0.55 (-0.44, 1.53)	7.67E-01
Bacteroidetes;Flavobacteriia;Flavobacteriales;Flavobacteriaceae;Capnocytophaga	75.7	141.59	171.86	-0.71 (-1.33, -0.10)	4.01E-01	-1.05 (-1.65, -0.44)	1.93E-02	-0.33 (-1.03, 0.37)	7.78E-01
Firmicutes; Clostridia; Clostridiales; Lachnospiraceae; Butyrivibrio	3.90	8.48	10.51	-1.04 (-1.93, -0.14)	4.01E-01	-1.26 (-2.15, -0.36)	5.81E-02	-0.22 (-1.21, 0.77)	9.01E-01
Proteobacteria;Gammaproteobacteria;Pasteurellales;Pasteurellaceae;Actinobacillus	17.96	11.02	88.26	0.44 (-0.60, 1.48)	8.06E-01	-1.73 (-2.78, -0.69)	2.08E-02	-2.17 (-3.31, -1.03)	9.90E-03
Spirochaetes;Spirochaetes;Spirochaetales;Spirochaetaceae;Treponema	346.73	201.42	131.28	0.92 (0.09, 1.75)	4.01E-01	1.44 (0.61, 2.27)	1.93E-02	0.52 (-0.41, 1.46)	7.67E-01
Synergistetes;Synergistia;Synergistales;Dethiosulfovibrionaceae;TG5	64.52	19.19	23.06	1.27 (0.33, 2.22)	4.01E-01	1.30 (0.35, 2.25)	5.81E-02	0.03 (-1.03, 1.08)	9.66E-01
Tenericutes;Mollicutes;Mycoplasmatales;Mycoplasmataceae;Mycoplasma	40.78	15.67	11.52	1.02 (0.02, 2.03)	4.94E-01	1.39 (0.37, 2.41)	5.81E-02	0.37 (-0.75, 1.48)	8.61E-01

[1] Only those taxa that have a significantly differential abundance with q<0.1 and a cook's distance <10 in at least one contrast are shown.
[2] Mean values refer to mean normalized counts of taxa according to each group.
[3] FDR adjusted p value. FDR adjustment was implemented at each level independently (i.e. class, order)

Supplementary Table S4. Differentially abundant taxa at all taxonomical levels in the contrasts between smokers versus nonsmokers and cotinine positive versus cotinine negative. Only those taxa that have a significantly differential abundance with q<0.1 and a cook's distance <10 in at least one contrast are shown.

Taxa[1]	Smokers			Cotinine positive vs. cotinine negative		
	Mean[2]	log2FC (CI)	q[3]	Mean[2]	log2FC (CI)	q[3]
Phylum						
Cyanobacteria	4.18	-1.65 (-1.32, 0.04)	2.42E-03	4.18	-1.64 (-2.63, -0.65)	3.11E-03
GN02	2.86	-0.89 (-1.34, -0.36)	1.24E-02	2.88	-0.83 (-1.47, -0.19)	2.46E-02
Proteobacteria	6440.96	-0.59 (-0.80, -0.37)	1.84E-05	6452.80	-0.65 (-0.91, -0.40)	8.50E-06
Spirochaetes	192.15	0.85 (0.47, 1.13)	6.95E-05	192.98	0.87 (0.46, 1.27)	1.07E-04
SR1	72.84	-0.74 (-1.04, -0.18)	1.24E-02	73.19	-0.94 (-1.48, -0.40)	2.11E-03
Synergistetes	27.49	1.21 (0.53, 1.32)	3.91E-06	27.62	1.13 (0.65, 1.61)	2.91E-05
Tenericutes	26.54	0.47 (0.00, 0.74)	7.82E-02	26.66	0.41 (-0.06, 0.88)	1.39E-01
Class						
Bacteroidetes; Flavobacteriia	272.99	-0.45 (-0.73, -0.27)	1.52E-03	273.46	-0.54 (-0.80, -0.28)	2.96E-04
Cyanobacteria; Chloroplast	5.01	-1.42 (-1.53, -0.13)	1.48E-03	5.00	-1.24 (-1.98, -0.49)	3.15E-03
Firmicutes; Erysipelotrichi	71.46	-0.29 (-0.35, 0.15)	9.21E-02	71.61	-0.28 (-0.57, 0.02)	1.26E-01
Fusobacteria;Fusobacteriia	1731.80	-0.12 (-0.28, 0.04)	1.99E-01	1733.59	-0.19 (-0.35, -0.03)	4.41E-02
GN02; BD1-5	3.38	-0.95 (-1.47, -0.45)	3.64E-03	3.39	-0.87 (-1.46, -0.28)	9.15E-03
Proteobacteria; Betaproteobacteria	2534.29	-0.87 (-1.26, -0.67)	2.75E-06	2542.93	-0.93 (-1.26, -0.59)	1.14E-06
Proteobacteria; Deltaproteobacteria	0.92	0.72 (0.10, 1.30)	8.30E-02	0.92	0.77 (0.10, 1.43)	4.94E-02
Proteobacteria; Epsilonproteobacteria	156.44	0.33 (0.18, 0.48)	7.71E-04	156.62	0.33 (0.16, 0.51)	6.62E-04
Proteobacteria; Gammaproteobacteria	3991.46	-0.49 (-0.72, -0.28)	7.71E-04	3981.69	-0.56 (-0.82, -0.30)	2.30E-04
Spirochaetes; Spirochaetes	208.45	0.70 (0.35, 1.03)	1.48E-03	209.14	0.71 (0.31, 1.11)	1.57E-03
Synergistetes; Synergistia	27.87	1.03 (0.40, 1.19)	5.99E-05	27.98	0.95 (0.49, 1.40)	2.96E-04
Tenericutes; RF3	0.55	0.79 (0.17, 1.56)	9.21E-02	0.55	0.66 (-0.08, 1.40)	1.36E-01
Order						
Actinobacteria; Actinobacteria; Bifidobacteriales	51.98	0.60 (0.30, 1.12)	1.96E-02	52.11	0.74 (0.27, 1.21)	3.94E-03
Bacteroidetes; Flavobacteriia; Flavobacteriales	260.33	-0.41 (-0.68, -0.24)	2.83E-03	261.01	-0.50 (-0.76, -0.25)	3.32E-04
Cyanobacteria; Chloroplast; Streptophyta	4.89	-1.32 (-1.56, -0.01)	1.40E-03	4.88	-1.31 (-2.10, -0.53)	2.22E-03
Firmicutes; Bacilli; Gemellales	1214.01	-0.45 (-0.61, -0.19)	8.82E-04	1215.36	-0.45 (-0.70, -0.21)	8.50E-04
Proteobacteria; Betaproteobacteria; Burkholderiales	175.49	-0.63 (-1.49, -0.71)	1.07E-02	175.65	-0.90 (-1.35, -0.45)	2.92E-04
Proteobacteria; Betaproteobacteria; Neisseriales	2175.67	-0.83 (-1.19, -0.60)	8.93E-06	2183.82	-0.86 (-1.19, -0.53)	9.77E-06
Proteobacteria; Deltaproteobacteria; Desulfobacterales	0.94	0.65 (0.11, 1.40)	9.14E-02	0.94	0.78 (0.09, 1.47)	4.80E-02
Proteobacteria; Epsilonproteobacteria; Campylobacterales	154.34	0.37 (0.21, 0.53)	2.21E-04	154.67	0.38 (0.20, 0.57)	2.11E-04
Proteobacteria; Gammaproteobacteria; Cardiobacteriales	19.39	-0.31 (-1.09, -0.46)	1.46E-01	19.45	-0.83 (-1.20, -0.47)	1.11E-04
Proteobacteria; Gammaproteobacteria; Pasteurellales	3713.13	-0.45 (-0.67, -0.25)	8.82E-04	3712.30	-0.50 (-0.75, -0.25)	2.92E-04
Spirochaetes; Spirochaetes; Spirochaetales	216.63	0.74 (0.42, 1.13)	8.82E-04	217.37	0.78 (0.37, 1.19)	4.84E-04
Synergistetes; Synergistia; Synergistales	28.58	1.01 (0.48, 1.30)	1.09E-04	28.68	1.01 (0.54, 1.47)	1.81E-04
Tenericutes; Mollicutes; Mycoplasmatales	19.93	1.07 (0.54, 1.45)	1.42E-04	20.00	1.03 (0.52, 1.54)	2.92E-04
Tenericutes; Mollicutes; RF39	9.23	-0.73 (-1.25, -0.20)	2.19E-02	9.27	-0.86 (-1.45, -0.27)	7.68E-03
Tenericutes; RF3; ML615J-28	0.61	0.84 (0.30, 1.86)	4.88E-02	0.62	0.85 (0.07, 1.63)	5.45E-02

[1] Only taxa that have a significantly differential abundance with q<0.1 and a cook's distance <10 in at least one contrast are shown.
[2] Mean values refer to mean normalized counts of taxa.
[3] FDR adjusted p value. FDR adjustment was implemented at each level independently (i.e. class, order)

Supplementary Table S4 continued.

Taxa[1]	Smokers			Cotinine positive vs. cotinine negative		
	Mean[2]	log2FC (CI)	q[3]	Mean[2]	log2FC (CI)	q[3]
Family						
Actinobacteria; Actinobacteria; Bifidobacteriales; Bifidobacteriaceae	53.51	0.60 (0.25, 1.07)	3.12E-02	53.65	0.73 (0.26, 1.21)	8.58E-03
Bacteroidetes; Bacteroidia; Bacteroidales; [Paraprevotellaceae]	792.15	0.42 (0.02, 0.51)	1.05E-02	794.85	0.36 (0.07, 0.64)	3.73E-02
Bacteroidetes; Bacteroidia; Bacteroidales; Porphyromonadaceae	907.68	-0.36 (-0.45, -0.02)	1.29E-02	910.38	-0.30 (-0.56, -0.04)	5.58E-02
Bacteroidetes; Flavobacteriia; Flavobacteriales; [Weeksellaceae]	115.81	-0.69 (-0.87, -0.31)	7.32E-05	116.17	-0.82 (-1.13, -0.51)	3.92E-06
Bacteroidetes; Flavobacteriia; Flavobacteriales; Flavobacteriaceae	146.38	-0.22 (-0.63, -0.15)	1.87E-01	146.64	-0.29 (-0.57, -0.01)	8.58E-02
Firmicutes; Bacilli; Gemellales; Gemellaceae	1221.43	-0.46 (-0.67, -0.24)	1.16E-03	1222.07	-0.48 (-0.74, -0.23)	8.40E-04
Firmicutes; Bacilli; Lactobacillales; Aerococcaceae	95.92	-0.76 (-0.87, 0.00)	7.53E-03	96.30	-0.67 (-1.16, -0.18)	2.35E-02
Firmicutes; Clostridia; Clostridiales; [Mogibacteriaceae]	117.12	0.28 (0.07, 0.53)	8.54E-02	117.49	0.28 (0.02, 0.55)	7.91E-02
Firmicutes; Clostridia; Clostridiales; Veillonellaceae	3322.06	0.24 (0.03, 0.43)	9.72E-02	3305.02	0.26 (0.03, 0.50)	6.32E-02
Fusobacteria; Fusobacteriia; Fusobacteriales; Fusobacteriaceae	966.04	-0.19 (-0.44, -0.10)	9.72E-02	967.65	-0.26 (-0.44, -0.07)	2.42E-02
Proteobacteria; Betaproteobacteria; Burkholderiales; Burkholderiaceae	184.31	-0.72 (-1.63, -0.81)	7.76E-03	184.37	-1.01 (-1.48, -0.55)	1.88E-04
Proteobacteria; Betaproteobacteria; Burkholderiales; Comamonadaceae	4.23	-0.84 (-1.44, -0.28)	2.10E-02	4.24	-0.96 (-1.58, -0.34)	8.66E-03
Proteobacteria; Betaproteobacteria; Neisseriales; Neisseriaceae	2203.57	-0.86 (-1.27, -0.67)	9.06E-06	2212.28	-0.91 (-1.25, -0.57)	3.92E-06
Proteobacteria; Deltaproteobacteria; Desulfobacterales; Desulfobulbaceae	0.93	0.74 (0.09, 1.38)	8.54E-02	0.93	0.79 (0.10, 1.48)	6.07E-02
Proteobacteria; Epsilonproteobacteria; Campylobacterales; Campylobacteraceae	154.42	0.37 (0.14, 0.47)	3.26E-04	154.71	0.37 (0.18, 0.56)	6.42E-04
Proteobacteria; Gammaproteobacteria; Cardiobacteriales; Cardiobacteriaceae	20.35	-0.35 (-1.21, -0.55)	1.37E-01	20.42	-0.92 (-1.30, -0.54)	2.96E-05
Proteobacteria; Gammaproteobacteria; Pasteurellales; Pasteurellaceae	3768.01	-0.44 (-0.70, -0.26)	2.52E-03	3762.33	-0.50 (-0.76, -0.24)	6.62E-04
Spirochaetes; Spirochaetes; Spirochaetales; Spirochaetaceae	207.72	0.81 (0.40, 1.10)	3.26E-04	208.49	0.80 (0.39, 1.20)	6.42E-04
Synergistetes; Synergistia; Synergistales; Dethiosulfovibrionaceae	28.50	1.12 (0.44, 1.26)	2.37E-05	28.62	1.00 (0.53, 1.47)	2.10E-04
Tenericutes; Mollicutes; Mycoplasmatales; Mycoplasmataceae	19.37	1.13 (0.47, 1.38)	7.32E-05	19.44	0.97 (0.46, 1.49)	8.40E-04
Genus						
Actinobacteria;Actinobacteria;Actinomycetales;Micrococcaceae;Rothia	1458.83	-0.33 (-0.64, -0.01)	1.23E-01	1456.86	-0.42 (-0.75, -0.08)	4.63E-02
Actinobacteria; Actinobacteria; Bifidobacteriales; Bifidobacteriaceae; Bifidobacterium	9.99	1.00 (0.70, 1.82)	5.65E-03	10.03	1.07 (0.45, 1.69)	3.45E-03
Bacteroidetes; Bacteroidia; Bacteroidales; Porphyromonadaceae; Paludibacter	25.55	0.49 (-0.02, 0.69)	5.83E-02	25.61	0.53 (0.12, 0.93)	4.41E-02
Bacteroidetes; Bacteroidia; Bacteroidales; Porphyromonadaceae; Porphyromonas	847.70	-0.57 (-0.62, -0.13)	9.90E-04	850.24	-0.50 (-0.79, -0.21)	3.45E-03
Bacteroidetes; Flavobacteriia; Flavobacteriales; Flavobacteriaceae; Capnocytophaga	145.64	-0.31 (-0.69, -0.20)	1.02E-01	145.92	-0.36 (-0.65, -0.07)	4.81E-02
Firmicutes; Bacilli; Gemellales; Gemellaceae; Gemella	21.50	-0.45 (-0.57, -0.02)	2.23E-02	21.48	-0.38 (-0.70, -0.05)	6.63E-02
Firmicutes; Clostridia; Clostridiales; Peptococcaceae; Peptococcus	13.21	-0.42 (-0.76, -0.04)	1.23E-01	13.24	-0.47 (-0.89, -0.06)	7.15E-02
Firmicutes; Clostridia; Clostridiales; Peptostreptococcaceae; Filifactor	28.16	0.54 (0.13, 1.03)	1.02E-01	28.27	0.65 (0.14, 1.17)	4.41E-02
Firmicutes; Clostridia; Clostridiales; Peptostreptococcaceae; Peptostreptococcus	86.83	-0.52 (-0.80, -0.14)	2.23E-02	87.16	-0.55 (-0.93, -0.17)	1.83E-02
Firmicutes; Clostridia; Clostridiales; Veillonellaceae; Megasphaera	170.35	0.32 (0.13, 0.78)	1.58E-01	169.92	0.45 (0.08, 0.81)	4.81E-02
Fusobacteria; Fusobacteriia; Fusobacteriales; Fusobacteriaceae; Fusobacterium	933.18	-0.28 (-0.50, -0.17)	1.52E-02	934.63	-0.33 (-0.52, -0.14)	3.45E-03
Proteobacteria; Betaproteobacteria; Burkholderiales; Burkholderiaceae; Lautropia	195.19	-0.80 (-1.64, -0.83)	3.51E-03	195.34	-1.04 (-1.51, -0.57)	2.52E-04
Proteobacteria; Betaproteobacteria; Neisseriales; Neisseriaceae; Eikenella	25.51	-0.29 (-0.89, -0.26)	2.28E-01	25.58	-0.59 (-0.96, -0.23)	7.43E-03
Proteobacteria; Betaproteobacteria; Neisseriales; Neisseriaceae; Neisseria	2176.51	-0.93 (-1.32, -0.66)	2.45E-05	2184.77	-0.97 (-1.34, -0.59)	1.23E-05
Proteobacteria; Deltaproteobacteria; Desulfobacterales; Desulfobulbaceae; Desulfobulbus	0.87	0.64 (0.04, 1.29)	1.32E-01	0.87	0.73 (0.05, 1.41)	9.27E-02
Proteobacteria; Epsilonproteobacteria; Campylobacterales; Campylobacteraceae; Campylobacter	145.87	0.28 (0.10, 0.41)	6.32E-03	146.14	0.30 (0.13, 0.47)	3.45E-03
Proteobacteria; Gammaproteobacteria; Cardiobacteriales; Cardiobacteriaceae; Cardiobacterium	20.11	-0.50 (-1.27, -0.61)	3.45E-02	20.16	-1.01 (-1.39, -0.63)	1.02E-05
Proteobacteria; Gammaproteobacteria; Pasteurellales; Pasteurellaceae; Aggregatibacter	327.12	-0.37 (-0.77, -0.20)	1.02E-01	328.07	-0.44 (-0.79, -0.10)	4.41E-02
Proteobacteria; Gammaproteobacteria; Pasteurellales; Pasteurellaceae; Haemophilus	3427.76	-0.52 (-0.76, -0.29)	1.02E-03	3423.53	-0.57 (-0.85, -0.29)	6.36E-04
Spirochaetes; Spirochaetes; Spirochaetales; Spirochaetaceae; Treponema	193.69	0.76 (0.37, 1.05)	9.90E-04	194.36	0.77 (0.38, 1.17)	1.29E-03
Synergistetes; Synergistia; Synergistales; Dethiosulfovibrionaceae; TG5	25.15	1.03 (0.40, 1.20)	8.66E-05	25.24	0.96 (0.51, 1.41)	4.67E-04
Tenericutes;Mollicutes;Mycoplasmatales;Mycoplasmataceae;Mycoplasma	18.07	0.96 (0.48, 1.44)	9.90E-04	18.14	0.91 (0.41, 1.42)	3.45E-03

[1] Only taxa that have a significantly differential abundance with q<0.1 and a cook's distance <10 in at least one contrast are shown.
[2] Mean values refer to mean normalized counts of taxa.
[3] FDR adjusted p value. FDR adjustment was implemented at each level independently (i.e. class, order)

Supplementary Table 5. DNA concentration in samples of sampling control

Sample ID	DNA concentration ng/µl
Saline sample 1	Below detectable levels
Saline sample 2	Below detectable levels
Mouthwash sample 1	7.52
Mouthwash sample 2	8.64

Supplementary Table 6a. Coefficient of variation (Phylum)

Batch	Number of samples	Replicates per sample	Diversity Measurement	QC sample 1	QC sample 2	QC sample 3
1	3	3	(Shannon Entropy)	2.32	5.52	0.88
			Firmicutes	4.18	4.38	1.78
			Bacteroidetes	3.17	10.79	2.03
			Proteobacteria	7.21	50.57	4.51
2	3	3	(Shannon Entropy)	1.65	2.02	5.95
			Firmicutes	1.02	3.36	5.36
			Bacteroidetes	4.78	4.20	12.95
			Proteobacteria	3.87	3.56	10.93

Supplementary Table 6b. Coefficient of variation (Genus)

Batch	Number of samples	Replicates per sample	Diversity Measurement	QC sample 1	QC sample 2	QC sample 3
1	3	3	(Shannon Entropy)	0.82	9.16	0.67
			Streptococcus	0.98	4.41	1.60
			Prevotella	3.07	16.07	3.07
			Haemophilus	6.32	38.12	3.02
2	3	3	(Shannon Entropy)	1.08	1.58	6.21
			Streptococcus	1.67	4.12	6.40
			Prevotella	7.99	2.63	9.01
			Haemophilus	9.60	3.51	6.26

Supplementary Figure S3. Richness and diversity comparisons between smokers (n=105) and non-smokers (n=225). (A) Richness (observed p=0.09), (B) Chao (p=0.41), (C) Simpson diversity index (p= 0.42). p values were obtained by implementing linear regression adjusting for age, gender and batch effects.

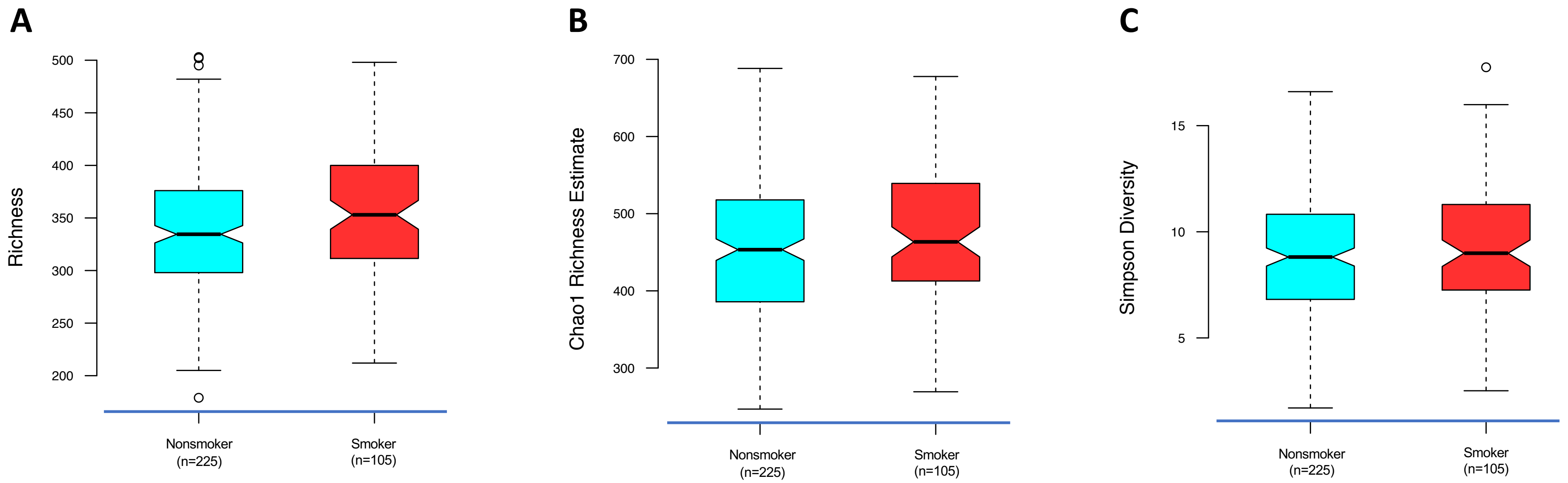

Supplement: Supplementary file 1 — Supplementary Information [file 41598_2018_29730_MOESM1_ESM.pdf]
